# Synergistic and Independent Actions of Multiple Terminal Nucleotidyl Transferases in the 3’ Tailing of Small RNAs in Arabidopsis

**DOI:** 10.1371/journal.pgen.1005091

**Published:** 2015-04-30

**Authors:** Xiaoyan Wang, Shuxin Zhang, Yongchao Dou, Chi Zhang, Xuemei Chen, Bin Yu, Guodong Ren

**Affiliations:** 1 State Key Laboratory of Genetic Engineering, Institute of Plant Biology and Department of Biochemistry, School of Life Sciences, Fudan University, Shanghai, China; 2 Center for Plant Science Innovation & School of Biological Sciences, University of Nebraska-Lincoln, Lincoln, Nebraska, United States of America; 3 Department of Botany and Plant Sciences & Institute of Integrative Genome Biology, University of California, Riverside, Riverside, California, United States of America; 4 Howard Hughes Medical Institute, University of California, Riverside, Riverside, California, United States of America; 5 Collaborative Innovation Center for Genetics and Development, School of Life Sciences, Fudan University, Shanghai, China; Pennsylvania State University, UNITED STATES

## Abstract

All types of small RNAs in plants, piwi-interacting RNAs (piRNAs) in animals and a subset of siRNAs in Drosophila and *C*. *elegans* are subject to HEN1 mediated 3’ terminal 2’-*O*-methylation. This modification plays a pivotal role in protecting small RNAs from 3’ uridylation, trimming and degradation. In Arabidopsis, HESO1 is a major enzyme that uridylates small RNAs to trigger their degradation. However, U-tail is still present in null *hen1 heso1* mutants, suggesting the existence of (an) enzymatic activities redundant with HESO1. Here, we report that UTP: RNA uridylyltransferase (URT1) is a functional paralog of HESO1. URT1 interacts with AGO1 and plays a predominant role in miRNA uridylation when HESO1 is absent. Uridylation of miRNA is globally abolished in a *hen1 heso1 urt1* triple mutant, accompanied by an extensive increase of 3’-to-5’ trimming. In contrast, disruption of URT1 appears not to affect the heterochromatic siRNA uridylation. This indicates the involvement of additional nucleotidyl transferases in the siRNA pathway. Analysis of miRNA tailings in the *hen1 heso1 urt1* triple mutant also reveals the existence of previously unknown enzymatic activities that can add non-uridine nucleotides. Importantly, we show HESO1 may also act redundantly with URT1 in miRNA uridylation when HEN1 is fully competent. Taken together, our data not only reveal a synergistic action of HESO1 and URT1 in the 3’ uridylation of miRNAs, but also independent activities of multiple terminal nucleotidyl transferases in the 3’ tailing of small RNAs and an antagonistic relationship between uridylation and trimming. Our results may provide further insight into the mechanisms of small RNA 3’ end modification and stability control.

## Author Summary

Small silencing RNAs are key regulators of gene expression in both plants and animals. HEN1-mediated 3’ terminal 2’-*O*-methylation plays a crucial role in small RNA stability control. In the absence of HEN1, several types of small RNAs become frequently uridylated (non-templated uridine addition) and trimmed, a phenomenon that is conserved across species. However, the underlying molecular mechanism is barely understood. In this study, we have discovered UTP: RNA uridylyltransferase (URT1) that acts synergistically with HESO1 in miRNA uridylation, in addition to its role in oligo-adenylated mRNA uridylation. Analyzing the miRNA profiles also reveals the existence of multiple terminal nucleotidyl transferases in the miRNA tailing process and an antagonistic action between uridylation and trimming. We believe this study will shed light on our understanding of how various terminal nucleotidyl transferases recognize their substrates and function coordinately.

## Introduction

MicroRNAs (miRNAs), a class of small non-coding RNAs with 20–24 nt in size, are master regulators of gene expression at post-transcriptional levels in both plants and animals [1,2]. They impact various biological processes such as development, metabolism and response to different biotic and abiotic stresses [1]. In Arabidopsis, miRNAs are typically derived from their precursor RNAs called primary miRNA transcripts (pri-miRNAs) through a sequential cleavage, from either loop proximal or loop distal, by DICER-LIKE 1 (DCL1) and its associated proteins [2–5]. After generation, the 3’ terminal riboses of miRNA/miRNA* duplexes are 2’-*O*-methylated by the RNA methyltransferase HUA ENHANCER1 (HEN1) [6]. One strand of the mature, methylated miRNA/miRNA* duplexes is selectively incorporated into their effector protein, mainly ARGONAUTE1 (AGO1), to form a silencing complex [7]. In the *hen1* mutants, miRNAs accumulate at lower levels and become heterogeneous in size, due to different levels of trimming and non-templated nucleotides addition (tailing, also commonly referred to as uridylation because uridine is preferentially added) at their 3’ ends [6,8]. Similar process also occurs to plant small interfering RNAs (siRNAs), animal PIWI-interacting RNAs (piRNAs) and a subset of animal siRNAs [9–16]. These results reveal an evolutionary conserved role of 3’ end methylation in protecting small RNAs from 3’ end tailing, trimming and degradation.

HEN1 SUPPRESSOR1 (HESO1) is a major enzyme responsible for the small RNA uridylation in Arabidopsis [17,18]. Loss-of-function mutations in *HESO1* result in reduced miRNA tailing length, increased miRNA abundance and partially restored morphological phenotypes of *hen1*. In contrast, over-expression of *HESO1* in *hen1* causes reduced miRNA accumulation and more severe morphological phenotypes. Collectively, these results demonstrate that uridylation triggers miRNA degradation [17,18]. Nevertheless, A substantial amount of miRNA uridylation is present in the null *hen1 heso1* mutants, suggesting the existence of additional small RNA uridylyltransferase(s) [17,18]. Besides HESO1, the Arabidopsis genome encodes nine additional terminal nucleotidyl transferases (TNTases) [17,18]. However, their involvement, if any, in miRNA uridylation is probably masked by HESO1 because none of their single mutants has any visible effect in terms of the restoration of *hen1* morphological phenotypes [18].

To identify additional miRNA uridylyltransferase (s), we screened for mutants with further recovered fertility in the *hen1-2 heso1-2* background. In this study, we show that a Proline-to-Leucine substitution at amino acid 618 (P618L) in URT1 increases the silique length and enhances the miRNA function of *hen1-2 heso1-2*. URT1 was previously identified as an enzyme that uridylates some mRNAs with short poly(A) tails and exhibits robust U-tailing activity *in vitro* [19]. We find that URT1 interacts with AGO1 and is responsible for miRNA, but not heterochromatic siRNA, uridylation in *hen1-2 heso1-2*. miRNA uridylation is globally abolished in the *hen1-2 heso1-2 urt1-3* mutant, accompanied by an extensive increase of trimming. These results demonstrate that HESO1 and URT1 act synergetically in miRNA uridylation and that trimming and tailing antagonize each other for the occupancy of miRNA 3’ ends. Moreover, a close investigation of the tailing status in *hen1-2 heso1-2 urt1-3* also reveals the addition of non-uridine nucleotides by yet uncharacterized TNTase(s), suggesting the complexity of miRNA tailing. Finally, we show HESO1 and URT1 may redundantly uridylate some miRNAs in the HEN1 competent background.

## Results

### A point mutation in URT1 increases the fertility of *hen1 heso1-2*

**Fig 1:**
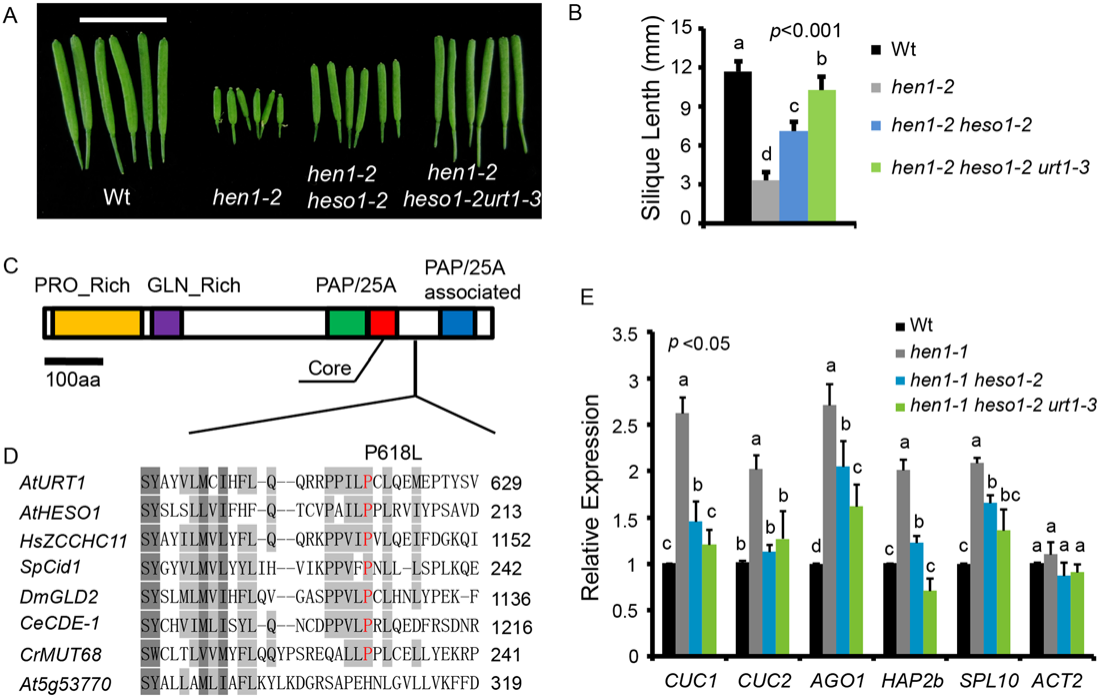
*urt1-3* increases the silique length in *hen1-2 heso1-2* and enhances miRNA function in *hen1-1 heso1-2*. (A) Fully-expanded siliques from plants of the indicated genotypes. Scale bar, 1cm. (B) Average silique length in indicated genotypes. 40 siliques from at least 6 individual plants for each genotype were analyzed. (C) Schematic structure of URT1 protein. PRO_Rich: Proline rich domain; GLN_Rich: Glutamine rich domain; PAP/25A: Poly A polymerase domain; Core: Core regions of PAP/25A; PAP/25A associated: Poly A polymerase associated domain. (D) Conservation of URT1 P618 across species. *At*, *A*. *thaliana*; *Hs*, *H*. *sapiens*; *Sp*, *S*. *pombe*; *Dm*, *D*. *melanogaster*; *Ce*, *C*.*elegans*; *Cr*, *C*. *reinhardtii*. (E) *urt1-3* enhances miRNA function in *hen1-1 heso1-2*. The transcript levels of five miRNA targets in indicated genotypes. Target mRNA accumulation in each genotype was quantified by qPCR using primers spanning the cleavage site and compared with those of wild type (Wt). The expression level of each gene in Wt was arbitrarily set to 1. Quantifications are normalized with *GAPDH* transcript. *ACT2* was served as an internal control. Values are means of three biological replicates ±SD.

To identify novel components in miRNA stability control, we performed another genetic screen in *hen1-2 heso1-2* (note that *hen1-2* is a weak allele and *heso1-2* is a null allele). Fertility, as reflected by the silique length, has been proved to be an effective indicator of miRNA activity in this system [17,20]. A mutant (m37-6) with markedly longer siliques than *hen1-2 heso1-2* was isolated from the M2 population of ethylmethanesulfonate (EMS) mutagenized *hen1-2 heso1-2*. The average silique length of m37-6 was ~1.0 cm, which was about 1.3 and 2.9 fold of those of *hen1-2 heso1-2* and *hen1-2*, respectively (Fig 1A and 1B). Backcross analyses revealed that the increased silique length phenotype was caused by a single recessive mutation. The mutation was roughly mapped to the marker nga168 on Chr. II, where HESO1 and two other TNTases (At2g40520 and At2g45620/URT1) were nearby this marker. Sequencing of these candidate genes revealed a single C-to-T nucleotide substitution, at the position +1853 relative to the translation start site (ATG) of URT1 (the mutation was hereafter referred to as *urt1-3*; *urt1-1* (Salk_087647C) and *urt1-2* (WISCDSLOXHS208_08D) were previously characterized to be defective in the uridylation of mRNAs with shortened poly(A) tails. [19]), which resulted in a Proline-to-Leucine change at amino acid 618 (P618L) (Fig 1C and 1D). P618 is located in an uncharacterized region that links the PAP domain and the PAP-associated domain. Blast analysis suggested that P618 is rather conserved, especially among those well-characterized TNTases (Fig 1D). Introduction of a genomic fragment of *URT1* fused with a *GFP* (*pURT1-URT1-GFP*) (S1 Fig) at its C-terminus into *hen1-2 heso1-2 urt1-3* restored its silique length to the *hen1-2 heso1-2* level, suggesting that the *urt1-3* mutation is responsible for the increased fertility of *hen1-2heso1-2*.

Introgression of the *urt1-3* mutation into *hen1-1 heso1-2* also increased its fertility (S2 Fig). Since *hen1-1* is a null allele of *hen1*, this observation suggested that the increase of *hen1 heso1-2* fertility by the *urt1-3* mutation doesn’t rely on the residual HEN1 activity. Morphological defects in *hen1* are mainly due to a reduction of miRNA activities and the null *heso1-2* mutation only partially restores the miRNA activities in *hen1* [17,18]. To determine whether the miRNA activities in *hen1-1 heso1-2* were further recovered by *urt1-3*, we compared the expression levels of five miRNA targets in L*er* (wild type, Wt), *hen1-1*, *hen1-1 heso1-2* and *hen1-1 heso1-2 urt1-3* by quantitative real-time RT-PCR (qPCR). Confirming our previous findings [17], the expression levels of all tested targets were increased in *hen1-1* as compared to those in Wt and their levels were partially restored in *hen1-1 heso1-2* (Fig 1E). Notably, the expression of these transcripts in *hen1-1 heso1-2 urt1-3* was further reduced, to the levels comparable to those in Wt (Fig 1E). Therefore, we conclude that the *urt1-3* mutation further enhances the miRNA function of *hen1-1 heso1-2*.

### Uridylation antagonizes 3’-to-5’ trimming of miRNAs

**Fig 2:**
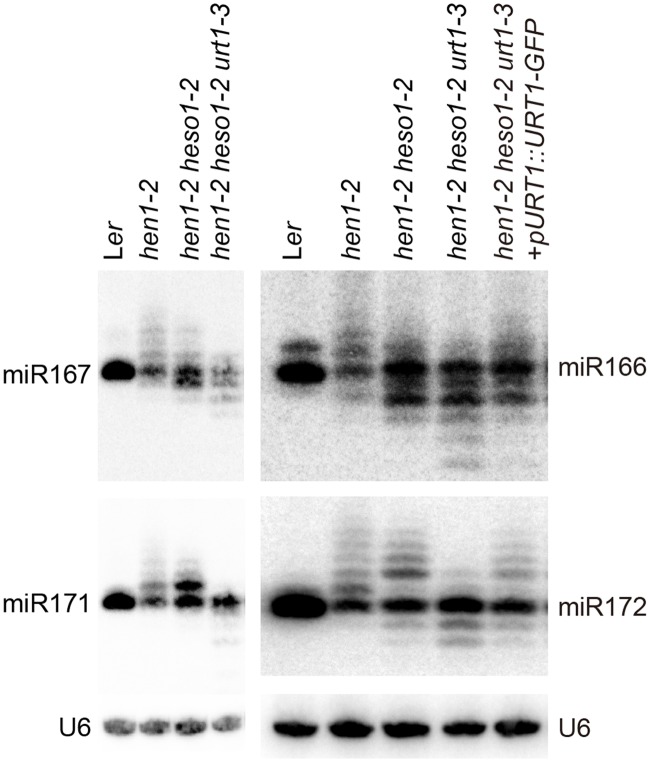
Small RNA northern blot analysis of miR167, miR166, miR171 and miR172 in various genotypes. U6 was served as a loading control.

We next examined the expression patterns of several miRNAs in Wt, *hen1-2*, *hen1-2 heso1-2* and *hen1-2 heso1-2 urt1-3* by the small RNA northern blot assay. We found that miRNA species with sizes longer than their annotated ones were barely detected in *hen1-2 heso1-2 urt1-3* for all the tested miRNAs (Fig 2). Instead, miRNAs in *hen1-2 heso1-2 urt1-3* became severely shortened in size as compared with those in *hen1-2* and *hen1-2 heso1-2* (Fig 2). The miRNA expression patterns of the genomic complementation line (*hen1-2 heso1-2 urt1-3* + *pURT1-URT1-GFP*) resembled those of *hen1-2 heso1-2*, demonstrating that the *urt1-3* mutation causes the changes of miRNA profiles in *hen1-2 heso1-2* (Fig 2).

Since unmethylated miRNAs can be frequently trimmed before uridylated [21], the size information given by the small RNA northern blot assay may only reflect the overall combination effect of trimming and tailing. In order to analyze the degree of uridylation reduction and trimming enhancement in detail, small RNA libraries with two biological replicates for each genotype were prepared from Wt, *hen1-2*, *hen1-2 heso1-2* and *hen1-2 heso1-2 urt1-3* and subject to the Illumina deep sequencing. Small RNA 3’ end modification analysis was performed according to a previous report with minor modifications (for details, see Materials and Methods) [18]. Based on the algorithm, small RNA reads were split into a 5’ genome matched component (5GMC) part and a 3’ tailed part, which reflects the degree of trimming and tailing, respectively. Only reads with 5GMC longer than 12nt were included in our analysis.

**Fig 3:**
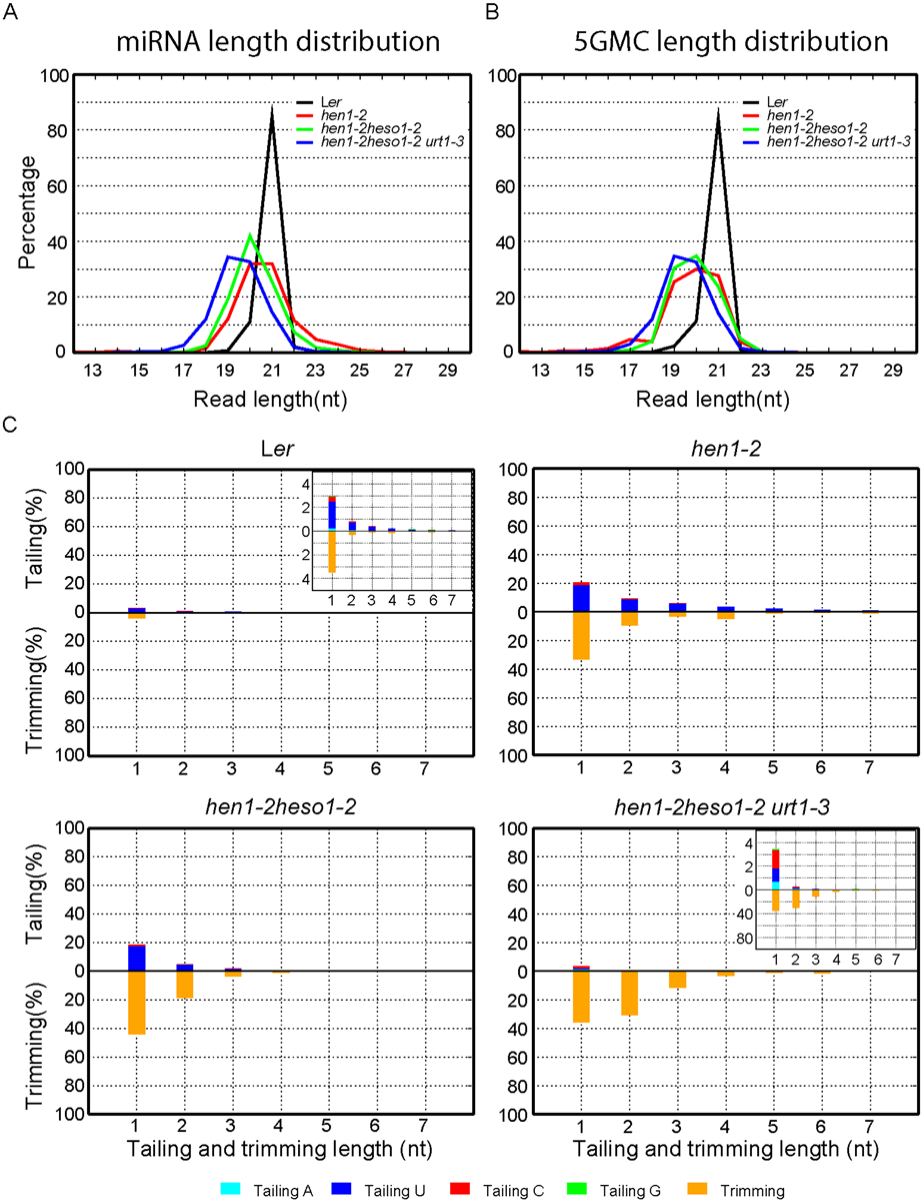
Global changes of miRNA profiles in *hen1-1 heso1-2 urt1-3*. (A) Size distribution of miRNAs in indicated genotypes. (B) Size distribution of 5GMC of miRNAs in indicated genotypes. 5GMC, 5’ genome matched component, see text for details. (C) Overall 3’ end signatures (including tail length, nucleotides addition composition and trimming extent) of miRNAs in indicated genotypes. The tailing and trimming extent was calculated as % (number of reads with indicated modification divided by total analyzed reads); the nucleotides addition composition was calculated as number of a nucleotide in the tail divided by tail length. Data from biological replicate 1 were shown. See S3 Fig online for data from biological replicate 2.

We first compared the overall length distribution of miRNAs among these four genotypes. As expected, about 84% of the miRNAs were 21nt in size in Wt. The size of miRNAs became heterogeneous in *hen1-2*, ranging from 18nt to 25nt (reads cutoff = 0.5%) with peaks at 20nt and 21nt (Fig 3A and S3A Fig). An obvious shift of this size range towards shorter ones was observed in *hen1-2 heso1-2* and became more prominent in *hen1-2 heso1-2 urt1-3* (Fig 3A and S3A Fig), which is consistent with our northern blot results (Fig 2). Notably, only less than 2.5% of the miRNAs were 22nt or longer in *hen1-2 heso1-2 urt1-3*, compared with ~9.1% of those in *hen1-2 heso1-2* (p<0.0001, F-test) (Fig 3A and S3A Fig). Moreover, the 5GMC pattern of *hen1-2 heso1-2 urt1-3* resembled the miRNA distribution pattern (Fig 3A and 3B and S3A and S3B Fig), indicating a drastic loss of tailing activity by the loss of functions in HESO1 and URT1. The distribution of 5GMC also revealed that a reduction of uridylation was accompanied by an increase of trimming (Fig 3B and S3B Fig). Indeed, both the levels and extents (more extensive truncation) of trimming were gradually increased in *hen1-2 heso1-2* and *hen1-2 heso1-2 urt1-3* when we quantified the degrees of tailing and trimming separately (Fig 3C and S3C Fig). Notably, a small peak of 5GMC at 17nt was observed in *hen1-2* (Fig 3B and S3B Fig), indicating a proportion of miRNAs favor to be trimmed to 17nt before they are further tailed. However, such a peak was not observed in either *hen1-2 heso1-2* or *hen1-2 heso1-2 urt1-3*, implicating a dynamic correlation between tailing and trimming.

**Fig 4:**
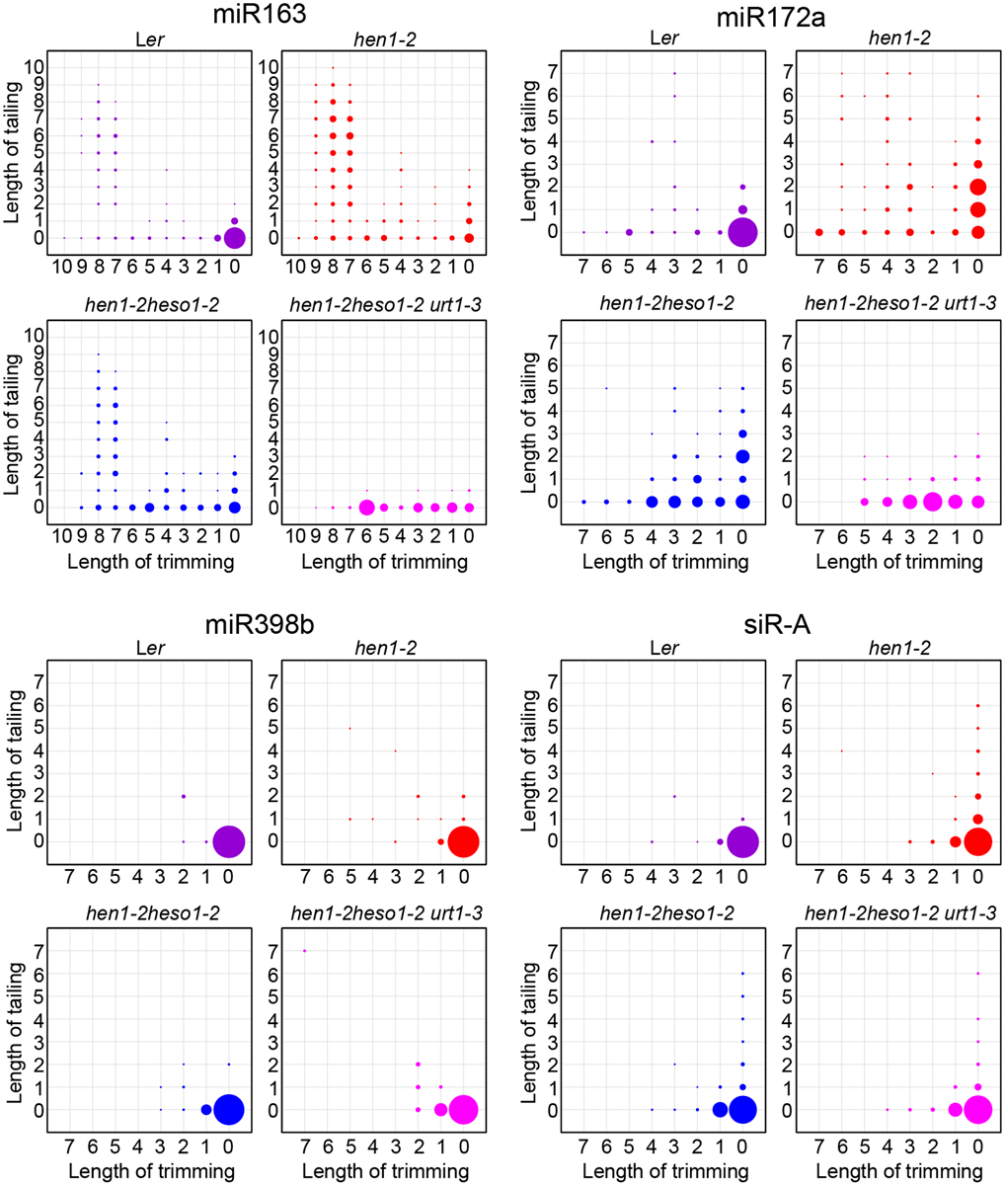
*urt1-3* affects miRNAs but not siRNAs uridylation in *hen1-2 heso1-2*. The X-axis represents the degree of trimming and the Y-axis represents the degree of tailing. The annotated miRNA sequences from miRBase v17.0 were served as standard sequences (i.e. these sequences are considered as non-tailed and non-trimmed.) For simplicity, we only analyzed reads started from the annotated 5’ ends. Thus, reads at coordinate position (0, 0) are exactly the same as annotated sequences and all reads from the same coordinate position are of same length. The relative abundance of each small RNA species is proportional to the diameter of the circles. Data from biological replicate 1 were shown. See S4 Fig online for more small RNAs and data from biological replicate 2.

Analyses of individual miRNAs further support our northern blot results and global miRNA profiles (Fig 2 and Fig 3). For most of the miRNAs analyzed, a gradual loss of uridylation activity was always accompanied by an increase of trimmed species and/or normal sized ones, albeit the degree of which varied among different miRNAs (Fig 4 and S4 Fig). Notably, long-tailed species could be readily detected in *hen1-2heso1-2* for some miRNAs (e.g. miR163 and miR164a) and the tails were almost abolished in *hen1-2heso1-2urt1-3* (Fig 4 and S4 Fig). More intriguingly, we found that several miRNAs (e.g. miR390 and miR398b) were resistant to both tailing and trimming modifications (Fig 4 and S4 Fig). The reason for this resistance is currently unknown.

### HESO1 and URT1 independent addition of non-uridine nucleotides by yet uncharacterized enzymes

Consistent with the overall length distribution (Fig 3A and S3A Fig), 3’ tailing was almost abolished in *hen1-2 heso1-2 urt1-3* (Fig 3C, S3C Fig and S1 Table). Intriguingly, we noticed a remarkably reduced preference for uridine (39.1%) when we calculated the nucleotide composition of the residual tails in *hen1-2 heso1-2 urt1-3*. As controls, the percentage of uridine addition in tails of Wt, *hen1-2* and *hen1-2 heso1-2* was 76.7%, 88.4% and 90.3%, respectively (Fig 3C, S3C Fig and S1 Table). Further analysis revealed that the raw counts of the other three types of nucleotides additions (i.e. adenosine, cytidine and guanosine) were comparable among *hen1-2*, *hen1-2 heso1-2* and *hen1-2 heso1-2 urt1-3*, although their relative proportions were increased in the triple mutant, due to an almost complete loss of uridylation (S1 Table). Based on our definition of tailing, a tiny proportion of tailed-only miRNAs might be alternatively introduced by the misprocessing, alternative processing and/or processing of pre-tailed pre-miRNAs [22,23]. We thus investigated miRNA species with 1nt trimmed and 1nt tailed, ensuring that the tails we analyzed were added after processing. We found that uridylation, but not other types of nucleotides addition were affected upon loss of function in HESO1 and URT1 (S2 Table). Taken together, these data unambiguously suggested that HESO1 and URT1 predominantly catalyze the uridylation of miRNAs and other TNTase(s) must exist to catalyze the non-uridine nucleotides addition with low activities.

### Uridylation of heterochromatic siRNA is largely unaffected by URT1

We also tested whether URT1, as like HESO1, plays a role in siRNA uridylation. A reduction in 3’ uridylation and/or an increase in 3’ end trimming were observed in several ta-siRNAs, suggesting that these ta-siRNAs are also recognized by URT1 (S4 Fig). Unexpectedly, the 3’ end modification pattern was largely unaffected in all three tested heterochromatic siRNAs (hc-siRNAs) in *hen1-2 heso1-2 urt1-3* as compared with those in *hen1-2 heso1-2* (Fig 4 and S4 Fig), which were shown to be substrates of HESO1 [18]. The enzyme(s) responsible for siRNA uridylation in *hen1heso1-2* awaits future characterization. In addition, our results may also suggest a differentiation of terminal uridyl transferases in substrate recognition.

### Uridylation of miRNAs by HESO1 and URT1 in HEN1^+^ background

**Fig 5:**
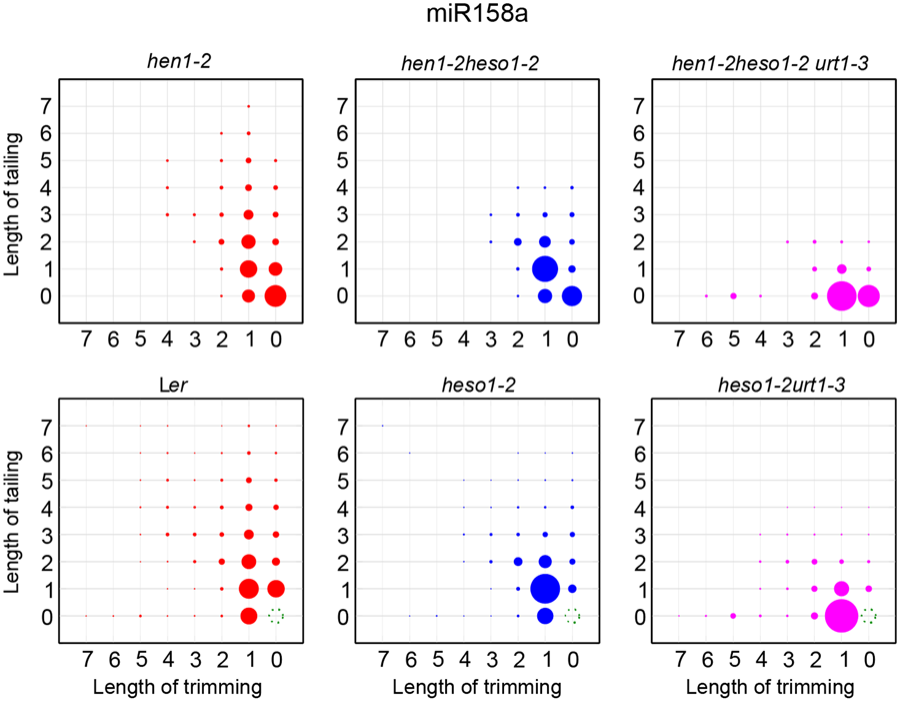
The tailing and trimming profiles of miR158a in L*er*, *heso1-2*, *heso1-2 urt1-3* resemble those in *hen1-2*, *hen1-2 heso1-2*, *hen1-2 heso1-2 urt1-3*. For L*er*, *heso1-2*, *heso1-2 urt1-3*, reads same as annotated miR158a sequence were removed and the rest of miR158a variants were renormalized to 100%. More interpretations of the matrix are depicted in Fig 4.

A small proportion of miRNAs in HEN1 competent background are unmethylated and these miRNAs are extensively uridylated and/or trimmed (Fig 5 and S5B Fig) [8,18,21]. We thus examined whether HESO1 and URT1 are catalyzers of miRNA uridylation in HEN1^+^ plants. To test this, *hen1-2 heso1-2 urt1-3* was crossed to *heso1-2* [17] and *heso1-2 urt1-3* was obtained from the F2 progeny by genotyping. Like *heso1-2*, *heso1-2 urt1-3* didn't show any morphological defects as compared with Wt under normal growth condition (S5A Fig) [17,24]. Small RNA deep sequencing reads ending with annotated miRNA sites were removed and the rest of miRNA variants were renormalized to 100% and subject to analysis as in Fig 4. We found the patterns for the loss of uridylation and the gain of trimming in L*er*, *heso1-2* and *heso1-2 urt1-3* resembled those observed in *hen1-2*, *hen1-2 heso11-2* and *hen1-2 heso1-2 urt1-3* (Fig 5 and S5B Fig), suggesting that HESO1 may act redundantly with URT1 in miRNA uridylation when HEN1 is fully competent.

### The P618L substitution impairs URT1 activity, affects its subcellular localization and diminishes its interaction with AGO1

**Fig 6:**
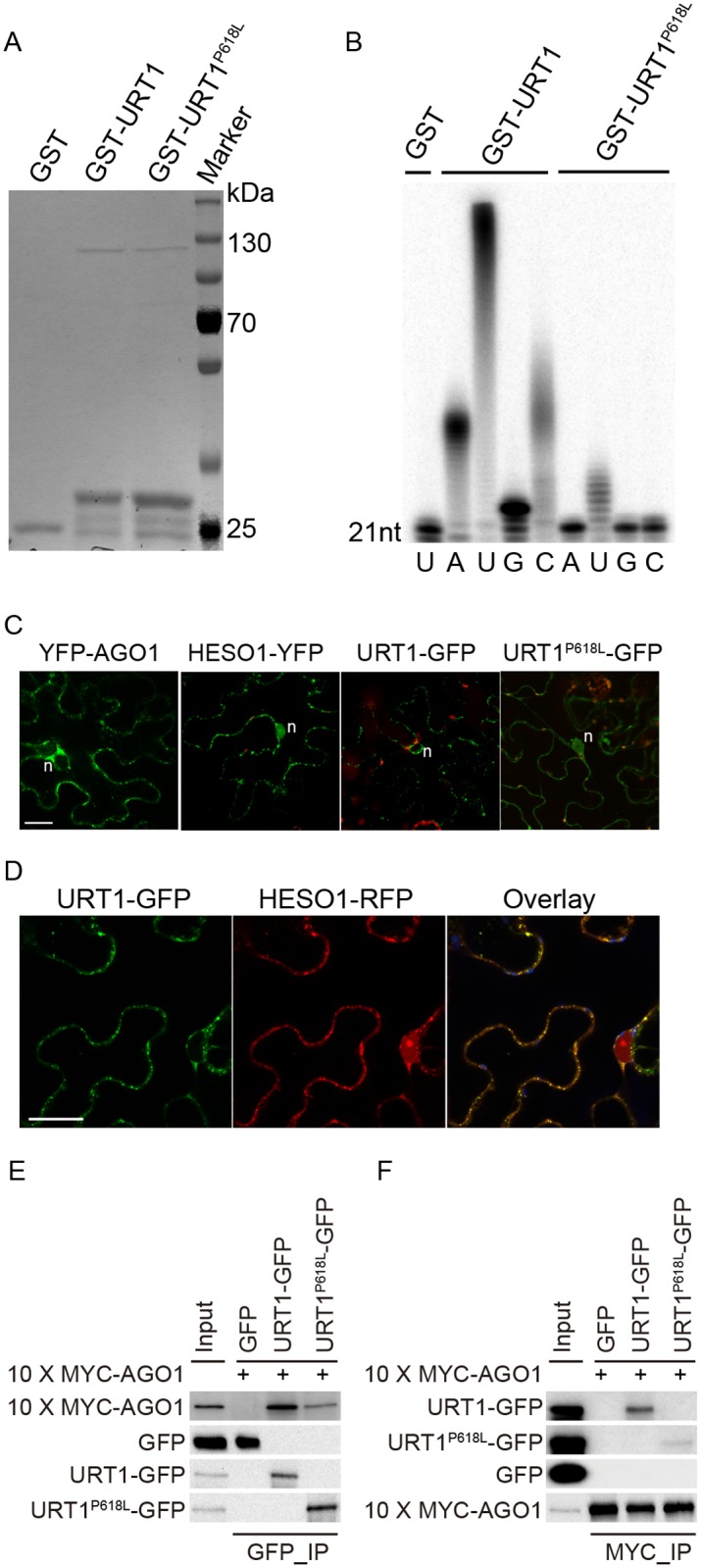
The P618L substitution impairs the URT1 activity, affects its subcellular localization and diminishes its interaction with AGO1. (A) Expression and purification of GST, GST-URT1 and GST-URT1^P618L^ in E.*coli* BL21(DE3). After purification, proteins were subject to SDS-polyacrylamide gel analysis and monitored by the comassie brillant blue staining. (B) Terminal nucleotidyl transferase activity of URT1 and URT1^P618L^. 5’ end P^32^ labeled miR165a were incubated with GST, GST-URT1 or GST-URT1^P618L^ in the presence of various nucleotide triphosphates for 30 minutes. After the reaction, RNAs were extracted and analyzed on a denaturing polyacrylamide gel. (C) Sub-cellular localization of YFP-AGO1, HESO1-YFP, URT1-GFP and URT1^P618L^-GFP. Note that only about 20% of YFP-AGO1 showed discrete cytoplasmic foci (C) while the remaining ones had a relatively even distribution[26]. For each construct, 40–50 cells were analyzed and a similar nucleus localization pattern was observed. n, nucleus. Scale bar, 20 μm. (D) URT1-GFP colocalized with HESO1-RFP. Paired constructs were coinfiltrated into N. *benthamiana* leaves. RFP and GFP fluorescence signals were monitored 40–48 h after infiltration by confocal microscopy. Scale bar, 20 μm. (E, F) Interactions between AGO1 and URT1 by the co-immunoprecipitation assay. 10xMYC-AGO1 and respective GFP tagged proteins were co-expressed in N. *benthamiana* leaves in the presence of P19 for 3 days and protein extracts were incubated with either anti-MYC-protein G-agarose beads or anti-GFP-protein G-agarose beads for 4 hrs to overnight. After reaction, proteins were resolved by the SDS-polyacrylamide gel electrophoresis and detected with respective antibodies. Input = 1%. (E) 10xMYC-AGO1 co-immunoprecipitates with URT1-GFP and URT1^P618L^-GFP. (F) URT1-GFP and URT1^P618L^-GFP co-immunoprecipitates with 10xMYC-AGO1.

Although the uridylyltransferase activity of URT1 on single stranded RNAs *in vitro* has been well documented [19,25], the effect of P618L substitution on its activity has not been determined and thus, how URT1^P618L^ increases the fertility in *hen1 heso1-2* and alters the miRNA profiles is unclear. To test this, we expressed both GST-URT1 and GST-URT1^P618L^ in *E*. *coli* BL21 and purified the recombinant proteins by the Glutathione Sepharose 4B beads (Fig 6A). Consistent with previous reports [19,25], GST-URT1 but not GST alone possessed a robust activity in incorporating UTP to the 3’ end of its RNA substrate (Fig 6B). In addition, GST-URT1 was also able to add a few adenosines (A) or cytidines (C) but only one to two guanosines (G) to its RNA substrate (Fig 6B). In contrast, only residual uridylyltransferase activities were detected for GST-URT1^P618L^ (Fig 6B), indicating the P618L single amino acid substitution severely impairs the URT1 activity without affecting its UTP preference.

We next examined the localization of URT1 by transient expression in *Nicotiana Benthamiana*. URT1-GFP was localized to the cytoplasmic foci, a pattern similar to those observed in HESO1 and AGO1 (Fig 6C) [24,26,27]. Indeed, we found that URT1-GFP colocalized with HESO1-RFP at the cytoplasmic foci (Fig 6D), supporting a role of URT1 in miRNA uridylation. While HESO1 was also targeted to the nucleus (100%, n = 43) (Fig 6C) [17], URT1-GFP signals could only be detected around the nuclear envelope but not in the nucleoplasm (100%, n = 53) (Fig 6C). Interestingly, URT1^P618L^-GFP signals were readily detected in the nucleoplasm (100%, n = 50) and the number of cytoplasmic foci was greatly reduced (n = 50) (Fig 6C), suggesting a change of localization of URT1 by the P618L single amino acid substitution. Western blot analysis suggested that the change of localization of URT1^P618L^-GFP might be not caused by passive diffusion of truncated URT1^P618L^-GFP proteins (S6C Fig). Bioinformatics prediction also failed to detect a loss of nuclear export signal (NES) /a gain of nuclear localization signal (NLS) by P618L.

Our recent study suggests that HESO1 uridylates AGO1-associated miRNAs through its interaction with AGO1 [24]. We thus asked whether URT1 also interacts with AGO1 and if so, whether URT1^P618L^ affects its interaction with AGO1. To test this, we transiently co-expressed URT1-GFP/ URT1^P618L^-GFP and 10xMYC-AGO1 in *N*. *benthamiana*. We were able to detect URT1-GFP in 10xMYC-AGO1 immunoprecipitates and *vice versa*, suggesting an interaction between URT1 and AGO1 (Fig 6E and 6F and S6 Fig). In contrast, GFP and 10xMYC-AGO1 could not co-immunoprecipitate with each other (Fig 6E and 6F and S6 Fig). Moreover, 10xMYC-DCL3 (aa1-280) failed to co-immunoprecipitate with URT1-GFP. These data suggest a direct interaction between URT1 and AGO1 (S6D Fig). The interaction between URT1-GFP and 10xMYC-AGO1 was not affected by the treatment of RNAse A (50 μg/ml), suggesting their association may be RNA-independent (S6D Fig). We also detect a positive, albeit weaker, interaction between URT1^P618L^-GFP and 10xMYC-AGO1 (Fig 6E and 6F and S6 Fig). However, we could not rule out the possibility that the diminution of protein-protein interaction was caused by the change of sub-cellular localization.

## Discussion

We have previously shown that HESO1 is a major enzyme responsible for the uridylation of all types of small silencing RNAs in *hen1* in Arabidopsis and also reveals the redundancy of small RNA uridylation [17,18]. Besides HESO1, the Arabidopsis genome encodes additional nine TNTases [17,18]. However, despite the conservation of PAP and/or PAP-associated domains, other sequences among these TNTases are highly divergent, which hinders the prediction of their exact function. Moreover, individually knock out of these nine TNTases in *hen1-8* fails to rescue the morphological phenotype of *hen1-8* [18]. In this study, we successfully identified URT1 as a functional homologue of HESO1 in triggering miRNA uridylation. In the accompanied paper, Tu et al also show that lack of URT1, but not other TNTases, results in an only minor reduction of uridylation of several miRNAs in *hen1-8* [28]. Taken together, these observations suggest that HESO1 and URT1 can compensate for each other to some extent and function synergistically.

A single point mutation in *URT1* results in the substitution of Proline by Leucine (P618L) in a yet uncharacterized region (Linker region) that bridges the poly(A) polymerase (PAP) domain and the PAP-associated domain (Fig 1C and 1D). Although P618L is rather conserved among several well-characterized TNTases (Fig 1D), sequence analysis reveals that only HESO1 and URT1 possess a complete “PAP-Linker-PAPA” structure among all ten TNTases in Arabidopsis (S3 Table), suggesting the importance of this structure for their proper function. As P618L diminishes URT1 activity without affecting its UTP preference *in vitro* (Fig 6B), it is likely that *urt1-3* is not a null allele. In agreement with this, we found that miRNA uridylation was not completely abolished in the *hen1-2 heso1-2 urt1-3* triple mutant (Fig 3C, S3C Fig and S1 Table). However, we cannot exclude the possibility that the residual miRNA uridylation is catalyzed by other uridydyl transferase(s) and *urt1-3* is null *in vivo*.

URT1 co-localizes with HESO1 and both enzymes interact with AGO1, consistent with their roles in the uridylation of AGO1-associated miRNAs [24,28]. However, unlike HESO1, URT1 is barely detectable in the nucleoplasm and may not affect heterochromatic siRNA uridylation in the *hen1heso1* background (Fig 6C, Fig 4 and S4 Fig). Moreover, URT1 appears to be less active than HESO1 *in vivo* since loss-of-function in URT1 alone has limited effects on the miRNA profile and growth of *hen1* [17,18,28]. However, we found that HESO1 and URT1 share very similar catalytic properties *in vitro* in terms of the UTP preference (Fig 6B) [17]. It shall be noted that an accurate comparison of their activities will require more strict conditions such as the removal of fusion tags. Moreover, our results also suggest that URT1 may be responsible for adding long tails to the 3’ ends of at least some miRNAs (e.g. miR163 and miR164a) *in vivo*. Alternatively, URT1 may trigger mono- or di-uridylation, which is required for additional tailing by yet uncharacterized TNTase(s). Future studies on their expression regulation and catalytic dynamics will help to understand their differences in the functional output.

Consistent with previous notion [17], the reduced levels of miRNA uridylation are accompanied by the increased levels and extents (more extensive truncation) of trimming, demonstrating that uridylation antagonizes trimming for the occupancy of 3’ ends of miRNAs (Fig 3B and S3B Fig). More interestingly, other types of nucleotides addition (C, A, G) become evident when uridylation was drastically reduced in the *hen1-2 heso1-2 urt1-3* triple mutant (Fig 3C, S3C Fig, and S1 and S2 Table), suggesting the involvement of additional TNTases in the miRNA tailing process. Indeed, these non-uridine nucleotides additions are largely unaffected by the loss of functions in HESO1 and URT1, albeit they occur at very low frequencies among different genotypes (S1 and S2 Table). Thus, the landscape of TNTases in small RNA 3’ end tailing is much more complicated than previously appreciated, although the biological relevance of non-uridine tailing modifications remains obscure.

What is the function of HESO1 and URT1 in Wt background? Although no visible morphological defects are detected in either *heso1-2* or *heso1-2 urt1-3* under normal growth condition, we show that uridylation of unmethylated miRNAs is greatly reduced in *heso1-2* and is almost abolished in *heso1-2 urt1-3* (Fig 5 and S5 Fig). Since uridylation triggers degradation of unmethylated miRNAs, it is possible that HESO1 and URT1 may play a role in the fine adjustment of miRNA abundance in Wt plants. In fact, it has been hypothesized that uridylation may act coordinately with SMALL RNA DEGRADING NUCLEASEs (SDNs), a class of 3’-to-5’ exonucleases that can remove methylated nucleotides, in the active turnover of miRNAs [29,30]. In addition, HESO1 and URT1 are likely involved in the clearance of 5’ cleavage products of miRNA guided slicing of target mRNAs. In fact, HESO1 together with one or more other TUTases can uridylates 5’ cleavage products and promotes their degradation [24]. Based on the action mode of URT1 in miRNA uridylation, it is plausible to speculate that URT1 is one of other enzyme(s) that catalyzes the uridylation of 5’ cleavage products. Moreover, URT1 has been shown to uridylate some mRNAs with short poly(A) tails [19], suggesting HESO1 and URT1 may have a broad spectrum of substrate RNAs *in vivo*. While the biological significance of the clearance or tentatively stabilization of their substrate RNAs through uridylation modification is unknown, one can simply envision that *heso1-2* and *heso1-2 urt1-3* plants are under “sub-healthy” conditions. Examining the effect of HESO1 and URT1 under various stress conditions will provide new clues for their functions.

## Materials and Methods

### Plant materials

All the Arabidopsis (*Arabidopsis thaliana*) strains used in this study are in the Landsberg *erecta* (L*er*) background unless otherwise indicated. *hen1-1*, *hen1-2*, *heso1-2*, *hen1-1 heso1-2* and *hen1-2 heso1-2* are previously described [17,18,31]. Ethyl methanesulfonate (EMS) mutagenesis in *hen1-2heso1-2* was conducted according to the Arabidopsis manual [32]. F2 population obtained from a cross between m37-6 and *hen1-8 heso1-1* (in the Columbia-0 background) [18] was used for the bulk segregation analysis. To obtain the *hen1-1 heso1-2 urt1-3* and *heso1-2 urt1-3* mutants, *hen1-2 heso1-2 urt1-3* was crossed with *hen1-1 heso1-2* and *heso1-2*, respectively. Individuals containing respective genotypes were identified in the F2 population. *hen1-1* and *hen1-2* were genotyped according to [31]. *urt1-3* was identified by digestion of the URT1dCAPSF/R PCR product with DdeI, which cut *urt1-3* but not Wt.

### Genomic complementation

For the genomic complementation assay, an approximately 4.2-kb DNA fragment containing the *URT1* coding region as well as 1.4 kb promoter region was amplified from L*er* genomic DNA using PrimeSTAR (Takara, R045A) with primers URT1gGWF/URT1gGWR and sub-cloned into an Entry vector pENTR-D-topo to produce pENTR-URT1g. The entry clone was transferred into the destination vector pMDC204 [33] by LR recombination to produce *pURT1*:*URT1-GFP*. The resulting plasmid was transformed into *hen1-2 heso1-2 urt1-3* via floral dipping [34] and hygromycin resistance was used for the transgenic plants selection.

### qPCR analysis and Small RNA northern blotting

qPCR analysis and small RNA northern blotting were performed as previously described [17,35].

### Small RNA library construction and deep sequencing

Total RNA extracted from inflorescence tissue of indicated genotypes was used for small RNA library construction. Small RNA libraries were prepared as indexed (i.e. individually bar-coded) and sequenced at 51bp single-end on an Illumina Hiseq2000 following the standard protocol. The small RNA sequence data were deposited to Gene Expression Omnibus (GEO) under the accession number GSE60826. Adaptor sequence was removed using Perl scripts. Analysis of small RNA 3’ end modification was performed according to [18]. The reference miRNA sequences were obtained from miRbase v17.0 (http://www.mirbase.org/). For the determination of 5GMC, the 5’ end of miRNA was locked (i.e. miRNAs with 5’ end different from the reference were removed from analysis) and no mismatch was permitted during the alignment. Nucleotides between the annotated 3’ end and 5GMC were considered as trimmed nucleotides, while all nucleotides downstream of 5GMC, regardless of templated or not, were considered as tailed.

### Terminal nucleotidyl transerases assay

*URT1* CDS was amplified by PCR with primers GST-URT1F/GST-URT1R and cloned into pGEX-4T-1 to generate a Glutathione-S-transferase (GST) tagged plasmid (GST-URT1). The *in vitro* enzymatic assay was conducted as previously described [17].

### Confocal microscopy

pH7-WGY-AGO1 (*pAGO1*:*YFP-AGO1*) [26], *35S*:*HESO1-YFP* [17]and *35S*:*HESO1-RFP* [24] were previously described. *pURT1*:*URT1* ^*P618L*^ *-GFP* was generated the same wasy as *pURT1*:*URT1-GFP* except that a *hen1-2heso1-2urt1-3* genomic DNA was used as a template. Respective constructs were expressed in *N*. *benthamiana* leaves for 40~48hr and subjected to imaging using a Nikon A1 confocal mounted on an Eclipse 90i Nikon compound microscope.

### Co-immunoprecipitation

The full-length *URT1* coding sequence (CDS) was amplified from a L*er* cDNA sample by PCR with primers URT1cGWF/URT1cGWR and sub-cloned into an Entry vector pENTR-D-topo to produce pENTR-URT1c. The entry clone was transferred into destination vectors pMDC83 [33] by LR recombination to produce 35S-URT1-GFP. 35S-URT1^P618L^-GFP was constructed the same way from a *hen1-2heso1-2urt1-3* cDNA sample. URT1-GFP/URT1^P618L^-GFP and 10xMYC tagged AGO1 (in pGWB521 backbone) [24,36] were co-expressed in the presence of P19 in *N*. *benthamiana* leaves for 72hr and harvested. Protein extraction and Co-immunoprecipitation analysis were performed as previously described [35]. Anti-GFP (Clontech, 632459) antibody pre-coupled to the protein G-agarose beads (Santa Cruz, sc-2002) and anti-c-MYC (Sigma, A7470) agarose beads were used to immunoprecipitate the GFP tagged and 10xMYC tagged proteins, respectively. Anti-GFP (Covance, MMS-118R) and Anti-MYC (Abgent, AM1007a) were used for the western blot assay.

### Bioinformatics

Online softwares cNLS Mapper (http://nls-mapper.iab.keio.ac.jp/cgi-bin/NLS_Mapper_form.cgi), NucPred (http://www.sbc.su.se/~maccallr/nucpred/cgi-bin/single.cgi) and NLStradamus (http://www.moseslab.csb.utoronto.ca/NLStradamus/) were used to predict the NLS peptide and NetNES (http://www.cbs.dtu.dk/services/NetNES/) was used to predict the NES peptide using URT1 and URT1^P618L^ as bait sequences.

### DNA and RNA Oligos

All the primers, LNA probes and other RNA/DNA oligos used in this study are listed in S4 Table.

## Supporting Information

S1 Fig Complementation of *hen1-2heso1-2urt1-3* by the URT1 genomic DNA. (A) Mature siliques from plants of the indicated genotypes. Bar = 1cm. Comp., *hen1-2heso1-2urt1-3* harboring the *URT1* genomic DNA (pMDC204-URT1g). Numbers indicates individual T2 lines. (B) Average silique length in various genotypes. For each genotype, 40 siliques from at least 6 plants were analyzed.
(PDF)

S2 Fig *urt1-3* increases the fertility of *hen1-1heso1-2*. (A) Mature siliques from plants of the indicated genotypes. Bar = 1cm. (B) Average silique length in various genotypes. For each genotype, 40 siliques from at least 6 plants were analyzed.
(PDF)

S3 Fig Global changes of miRNA profiles in *hen1-1heso1-2urt1-3* (Biological replicate 2), related to Fig 3. (A) Size distribution of miRNAs in indicated genotypes. (B) Size distribution of 5GMC of miRNAs in various genotypes. 5GMC, 5’ genome matched component, see text for details. (C) Overall 3’ end signatures (including tail length, nucleotides composition and trimming extent) of miRNAs in indicated genotypes.
(PDF)

S4 Fig *urt1-3* affects miRNAs but not siRNAs uridylation in *hen1-2heso1-2*, related to Fig 4. The X-axis represents the degree of trimming and the Y-axis represents the degree of tailing. The annotated miRNA sequences from miRBase v17.0 are served as standard sequences (i.e. these sequences are considered as non-tailed and non-trimmed.) For simplicity, we only analyzed reads started from the annotated 5’ ends. Thus, reads at coordinate position (0,0) are exactly same as annotated ones and all reads at same coordinate position are of same length. The relative abundance of each small RNA species is proportional to the diameter of the circles.
(PDF)

S5 Fig HESO1 and URT1 uridylates unmethylated miRNAs in HEN1/+ background, related to Fig 5. (A), Morphological phenotypes of L*er*, *heso1-2* and *heso1-2urt1-3*. Bar = 1cm. (B) The tailing and trimming status of seven miRNAs in L*er*, *heso1-2* and *heso1-2urt1-3*. Reads same as annotated miRNA sequences were removed and the rest of miRNA variants were renormalized to 100%. More interpretations of the matrix are depicted in S4 Fig.
(PDF)

S6 Fig Interactions between AGO1 and URT1 by the co-immunoprecipitation assay, related to Fig 6. Input = 1%. IB, immunoblot. IP, Immunoprecipitation. (A) 10xMYC-AGO1 co-immunoprecipitates with URT1-GFP and URT1^P618L^-GFP. Red box, regions shown in Fig 6E. (B) URT1-GFP and URT1^P618L^-GFP co-immunoprecipitates with 10xMYC-AGO1. Red box, regions shown in Fig 6F. (C) Transient expression of URT1-GFP and URT1^P618L^-GFP in *Nicotiana Benthamiana*. (D) The interaction between 10xMYC-AGO1 and URT1-GFP is RNA independent. Arrow, URT1-GFP or URT1^P618L^-GFP. Star, 10xMYC-AGO1. Pound, 10xMYC-DCL3 (aa1-280).
(PDF)

S1 Table Tailing status and nucleotides composition in each genotype. (XLSX)

S2 Table Tailing status and nucleotides composition with coordinate positions (1, 1). (XLSX)

S3 Table Conservation of P motif among ten terminal nucleotidyl transferases in Arabidopsis. Note that only HESO1 and URT1 possess all three signatures.
(PDF)

S4 Table List of primers and oligos in this study. (DOCX)
