# Supplementary figures and images for: Synergistic and Independent Actions of Multiple Terminal Nucleotidyl Transferases in the 3’ Tailing of Small RNAs in Arabidopsis
Source: PLoS Genet. 2015 Apr 30;11(4):e1005091. doi: 10.1371/journal.pgen.1005091 (PMC4415790; doi:10.1371/journal.pgen.1005091)

S1 Figure

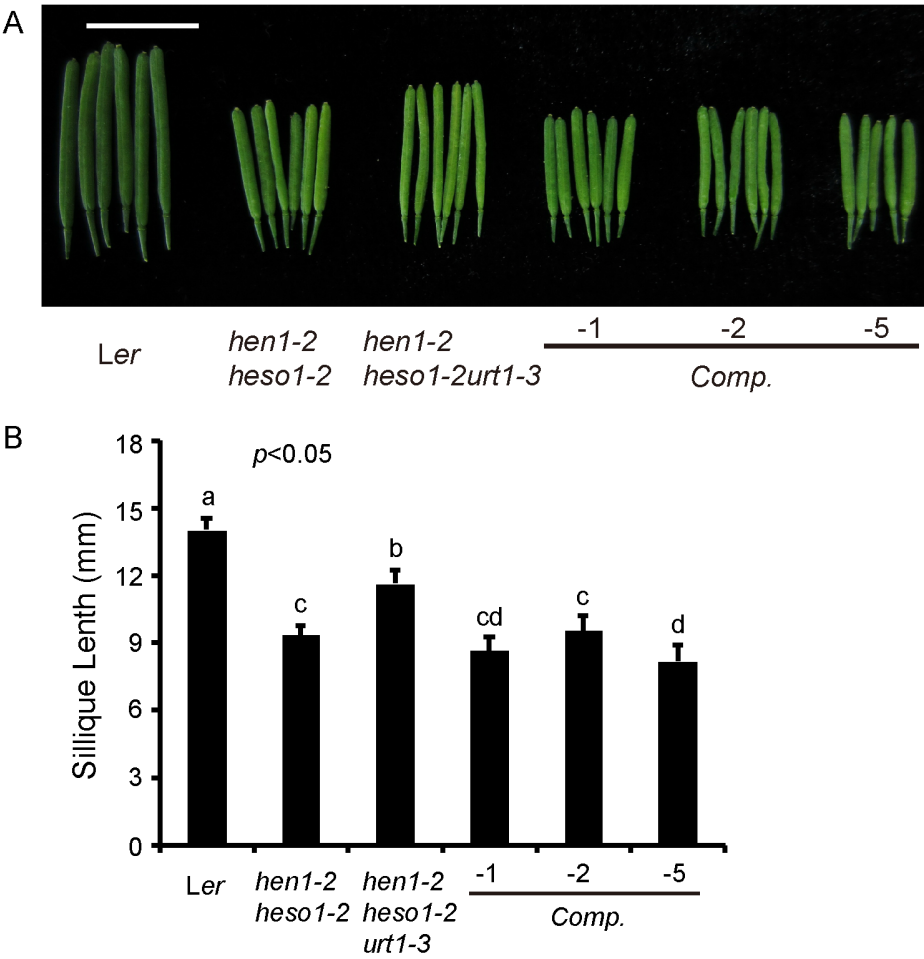

Supplement: S1 Fig — (A) Mature siliques from plants of the indicated genotypes. Bar = 1cm. Comp., hen1-2heso1-2urt1-3 harboring the URT1 genomic DNA (pMDC204-URT1g). Numbers indicates individual T2 lines. (B) Average silique length in various genotypes. For each genotype, 40 siliques from at least 6 plants were analyzed. (PDF) [file pgen.1005091.s001.pdf]

S2 Figure

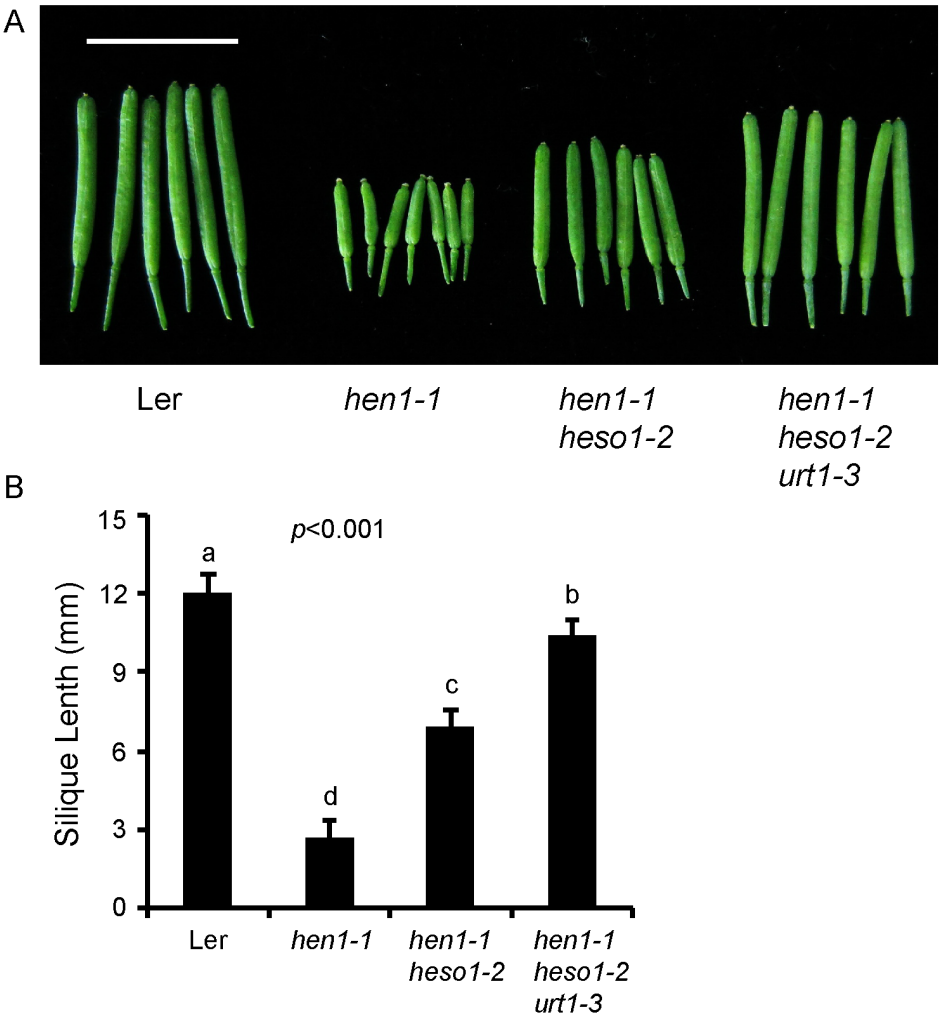

Supplement: S2 Fig — (A) Mature siliques from plants of the indicated genotypes. Bar = 1cm. (B) Average silique length in various genotypes. For each genotype, 40 siliques from at least 6 plants were analyzed. (PDF) [file pgen.1005091.s002.pdf]

S3 Figure

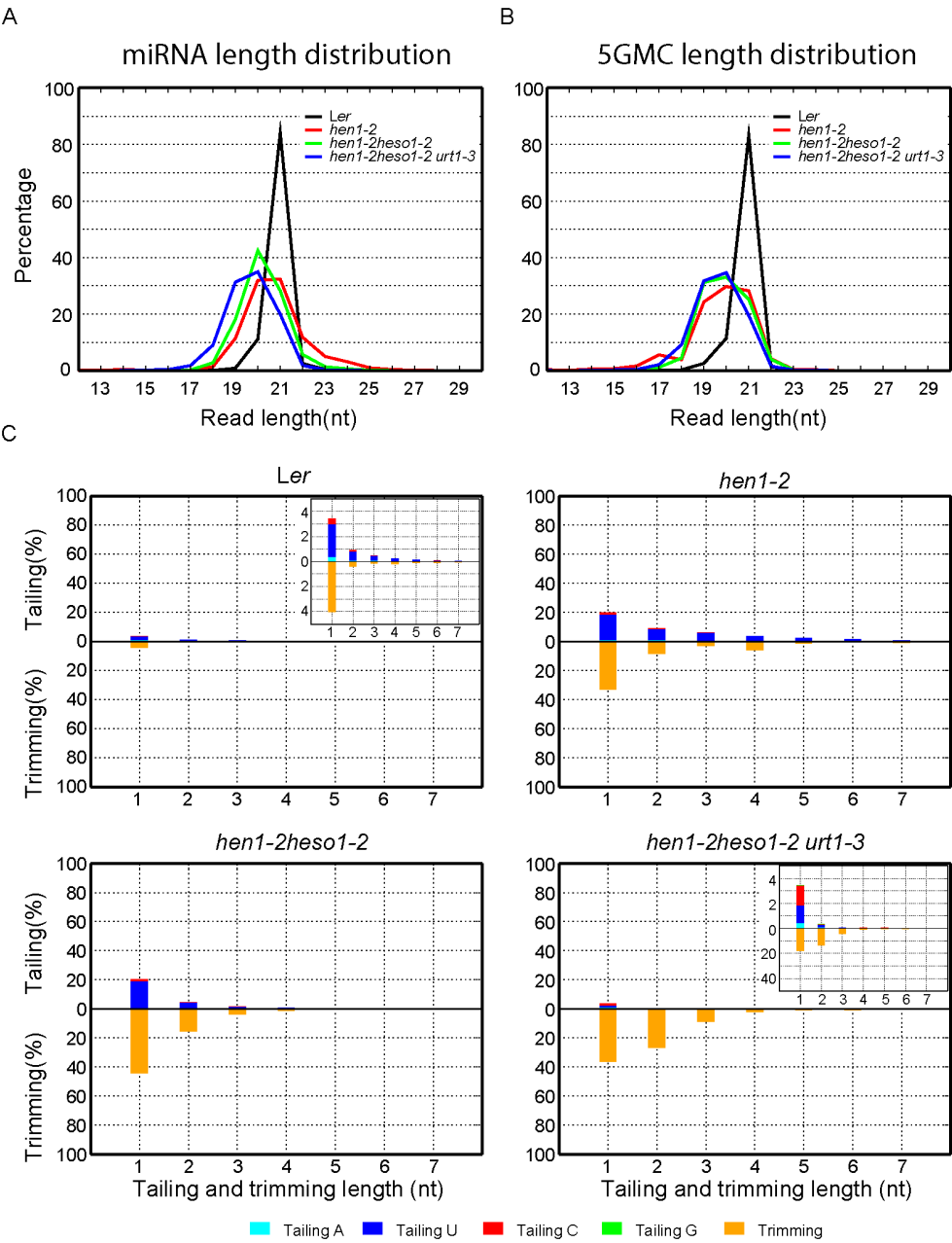

Supplement: S3 Fig — (A) Size distribution of miRNAs in indicated genotypes. (B) Size distribution of 5GMC of miRNAs in various genotypes. 5GMC, 5’ genome matched component, see text for details. (C) Overall 3’ end signatures (including tail length, nucleotides composition and trimming extent) of miRNAs in indicated genotypes. (PDF) [file pgen.1005091.s003.pdf]

S5 Figure

A

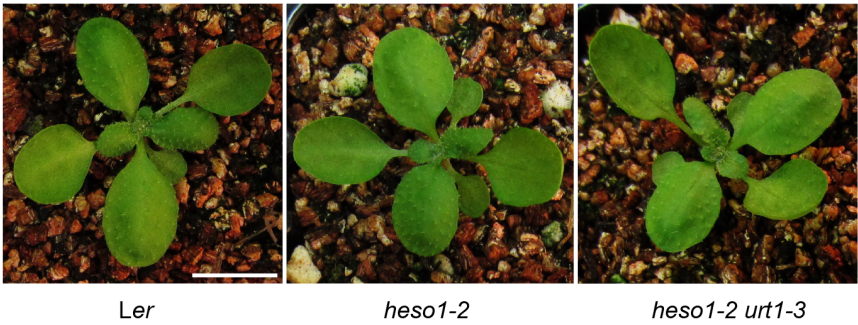

B

miR156a

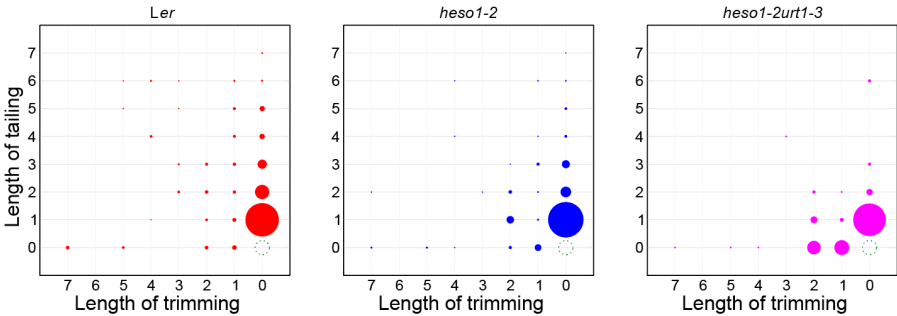

Re\_miR156a

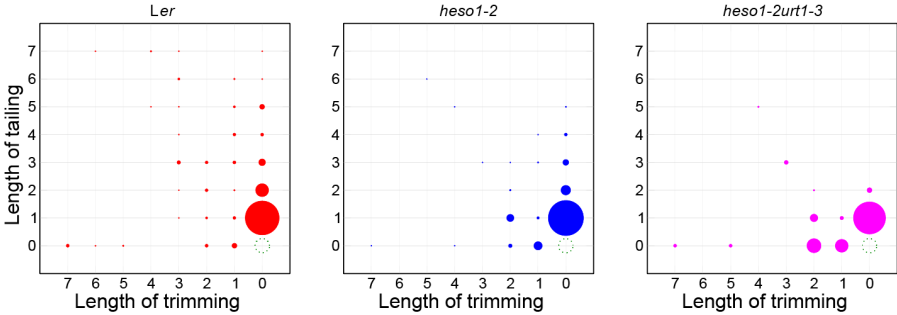

miR158a

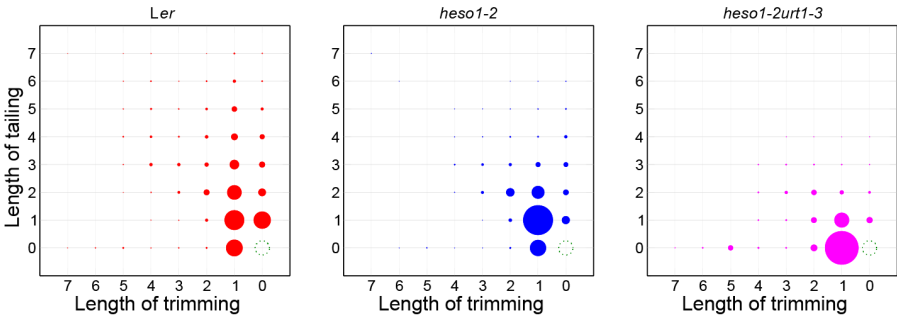

Re\_miR158a

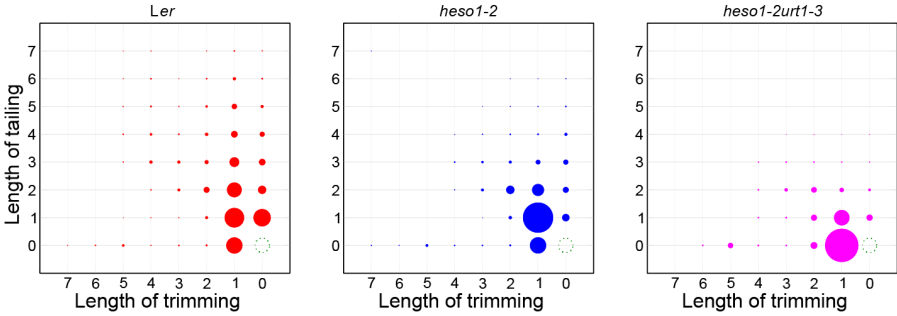

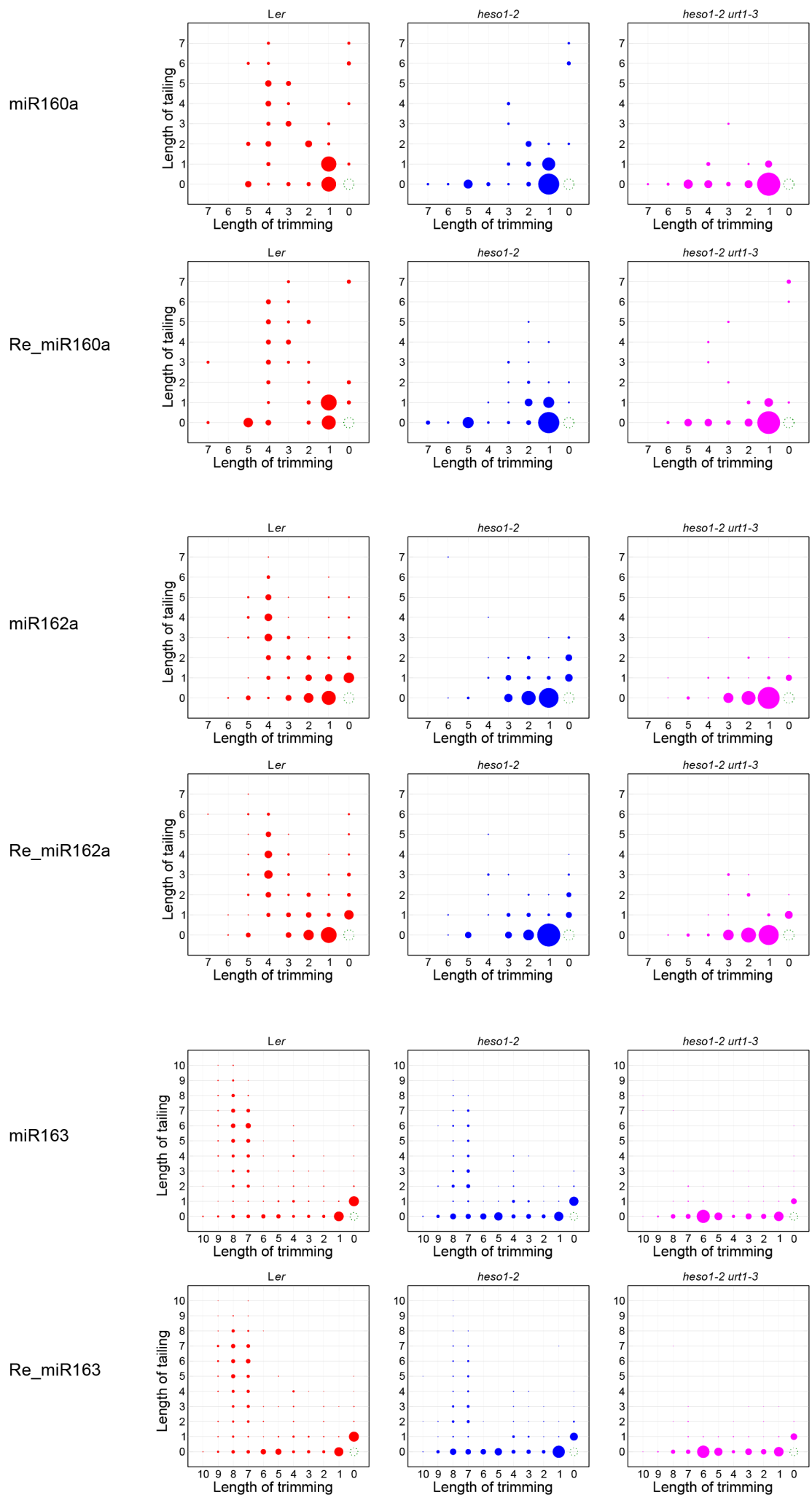

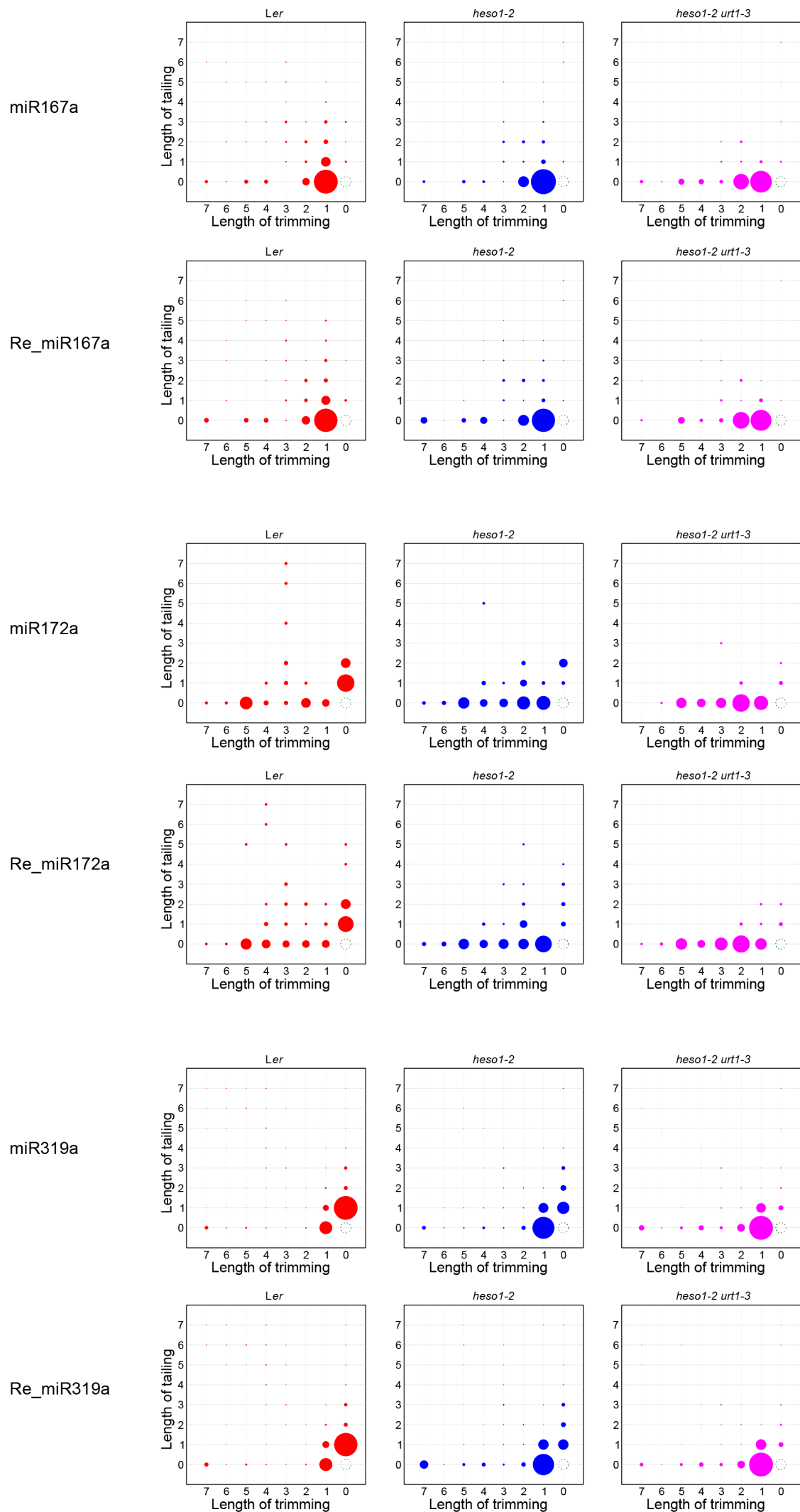

Supplement: S5 Fig — (A), Morphological phenotypes of Ler, heso1-2 and heso1-2urt1-3. Bar = 1cm. (B) The tailing and trimming status of seven miRNAs in Ler, heso1-2 and heso1-2urt1-3. Reads same as annotated miRNA sequences were removed and the rest of miRNA variants were renormalized to 100%. More interpretations of the matrix are depicted in S4 Fig. (PDF) [file pgen.1005091.s005.pdf]

S6 Figure

A IP: @GFP, related to Figure 6E

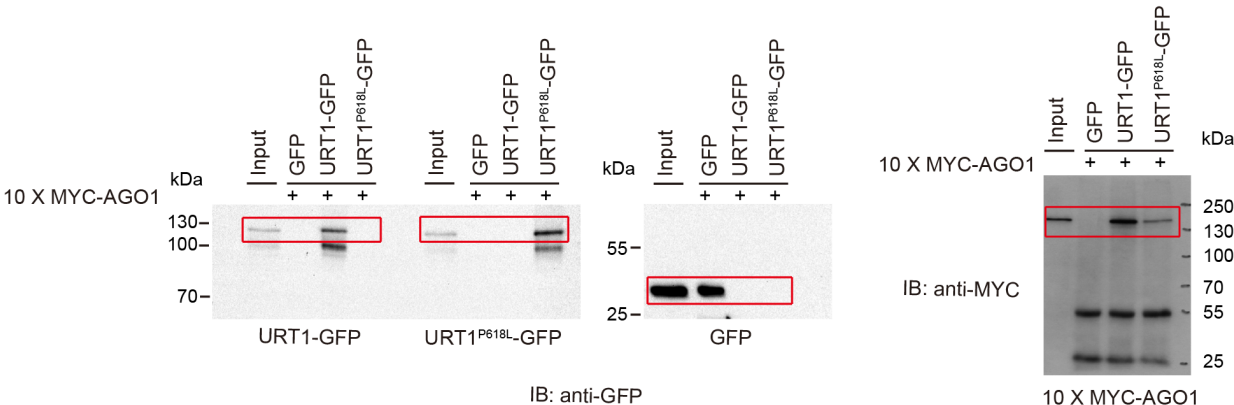

B IP: @MYC, related to Figure 6F

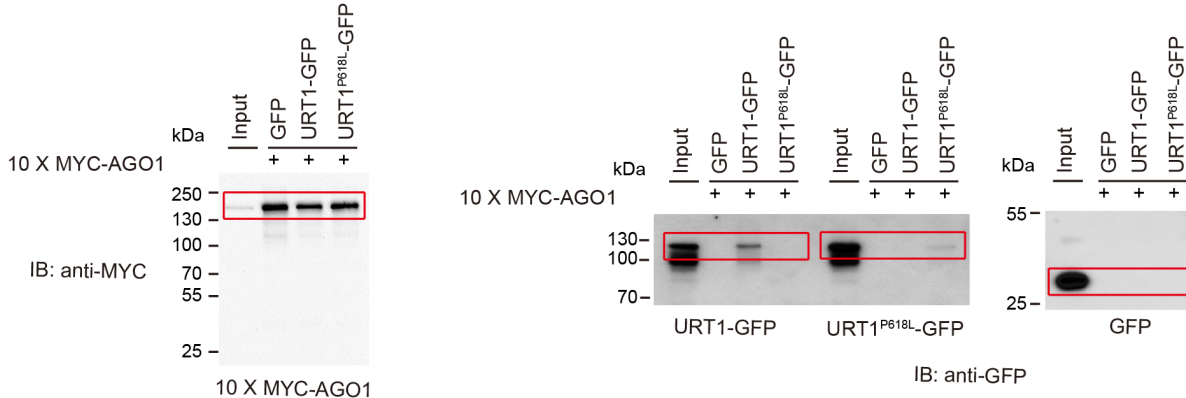

C

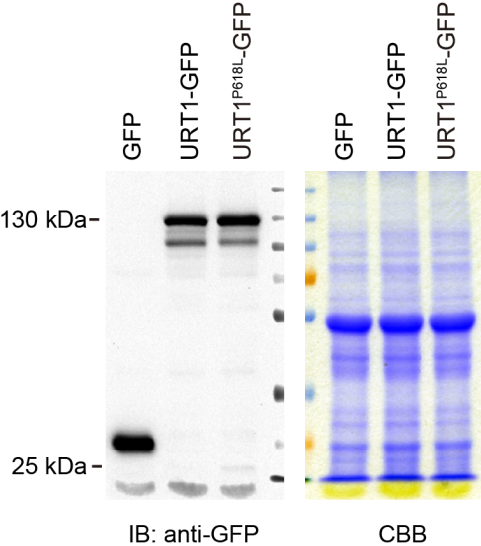

D

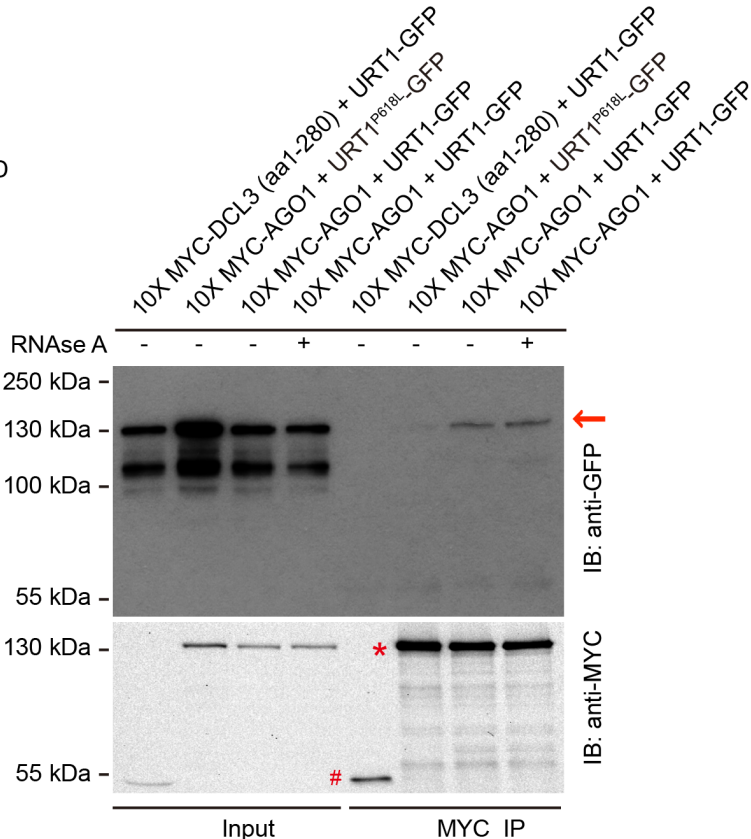

Supplement: S6 Fig — Input = 1%. IB, immunoblot. IP, Immunoprecipitation. (A) 10xMYC-AGO1 co-immunoprecipitates with URT1-GFP and URT1P618L-GFP. Red box, regions shown in Fig 6E. (B) URT1-GFP and URT1P618L-GFP co-immunoprecipitates with 10xMYC-AGO1. Red box, regions shown in Fig 6F. (C) Transient expression of URT1-GFP and URT1P618L-GFP in Nicotiana Benthamiana. (D) The interaction between 10xMYC-AGO1 and URT1-GFP is RNA independent. Arrow, URT1-GFP or URT1P618L-GFP. Star, 10xMYC-AGO1. Pound, 10xMYC-DCL3 (aa1-280). (PDF) [file pgen.1005091.s006.pdf]
