# Supplementary material for: Synergistic and Independent Actions of Multiple Terminal Nucleotidyl Transferases in the 3’ Tailing of Small RNAs in Arabidopsis
Source: PLoS Genet. 2015 Apr 30;11(4):e1005091. doi: 10.1371/journal.pgen.1005091 (PMC4415790; doi:10.1371/journal.pgen.1005091)
Supplement: S4 Fig — The X-axis represents the degree of trimming and the Y-axis represents the degree of tailing. The annotated miRNA sequences from miRBase v17.0 are served as standard sequences (i.e. these sequences are considered as non-tailed and non-trimmed.) For simplicity, we only analyzed reads started from the annotated 5’ ends. Thus, reads at coordinate position (0,0) are exactly same as annotated ones and all reads at same coordinate position are of same length. The relative abundance of each small RNA species is proportional to the diameter of the circles. (PDF) [file pgen.1005091.s004.pdf]

S4 Figure

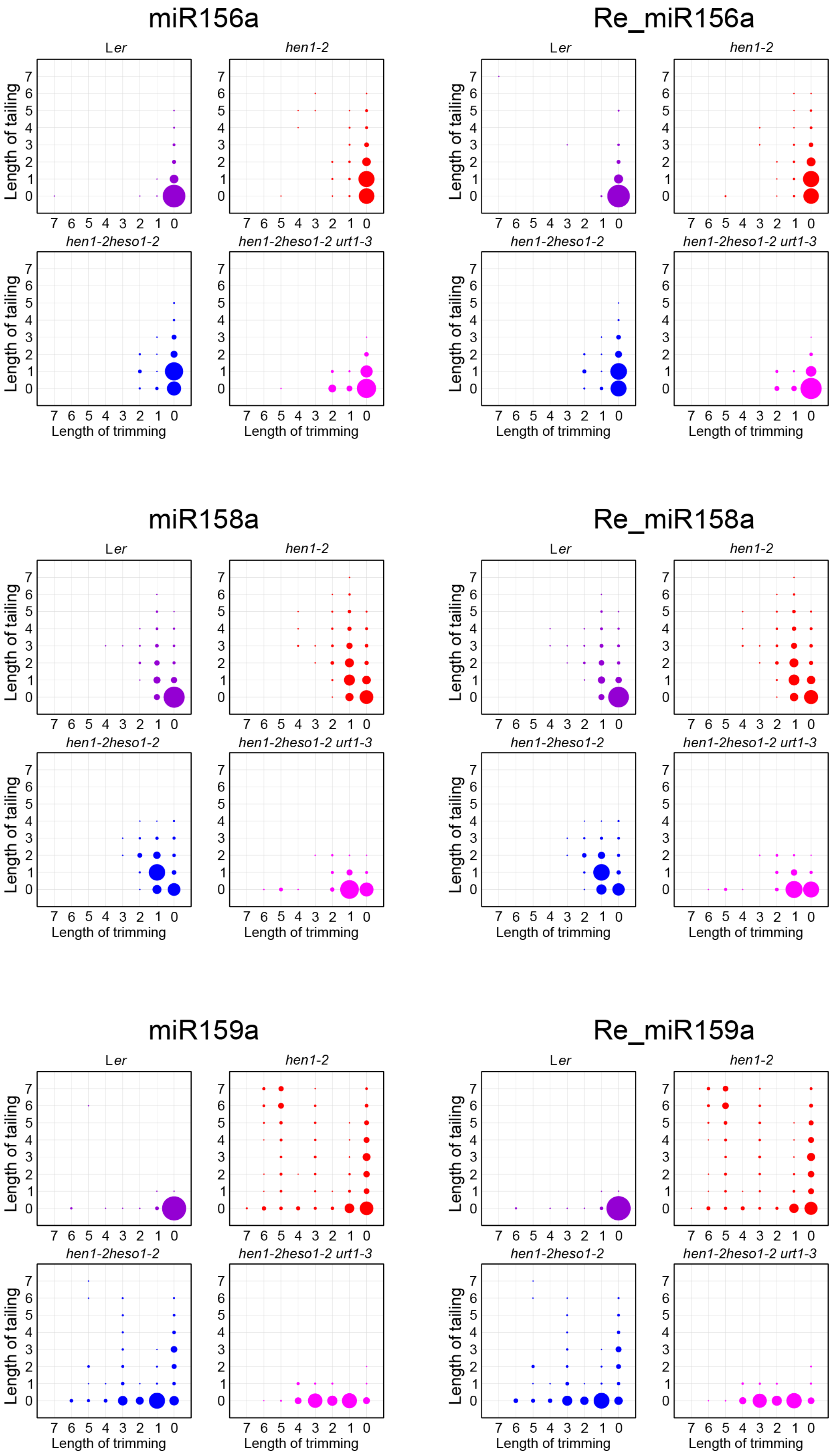
miR156a
Re_miR156a
miR158a
Re_miR158a
miR159a
Re_miR159a
Ler
hen1-2
hen1-2heso1-2
hen1-2heso1-2 urt1-3
Length of tailing
Length of trimming

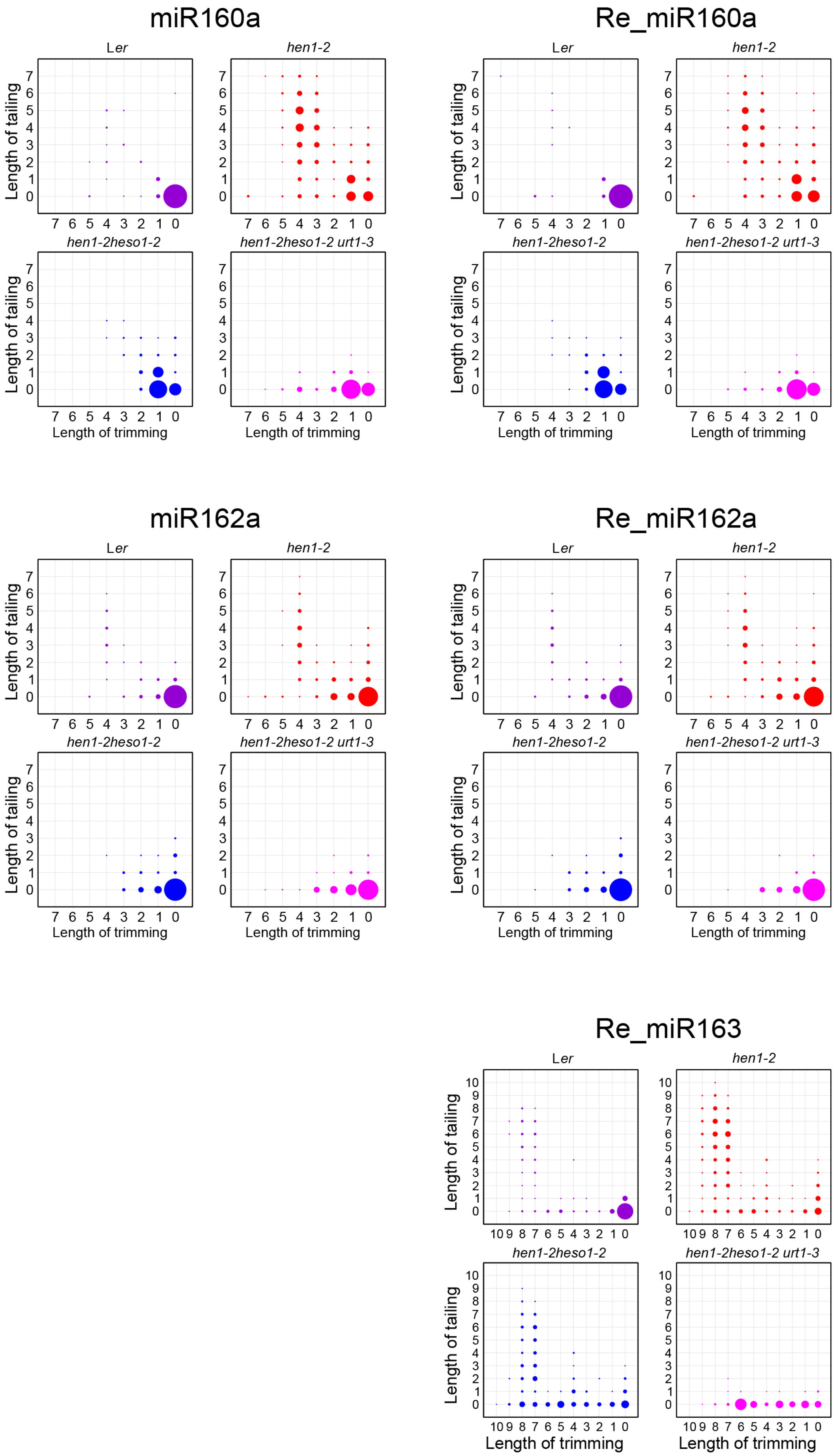
miR160a
Re_miR160a
miR162a
Re_miR162a
Re_miR163
Ler
hen1-2
hen1-2heso1-2
hen1-2heso1-2 urt1-3
Length of tailing
Length of trimming

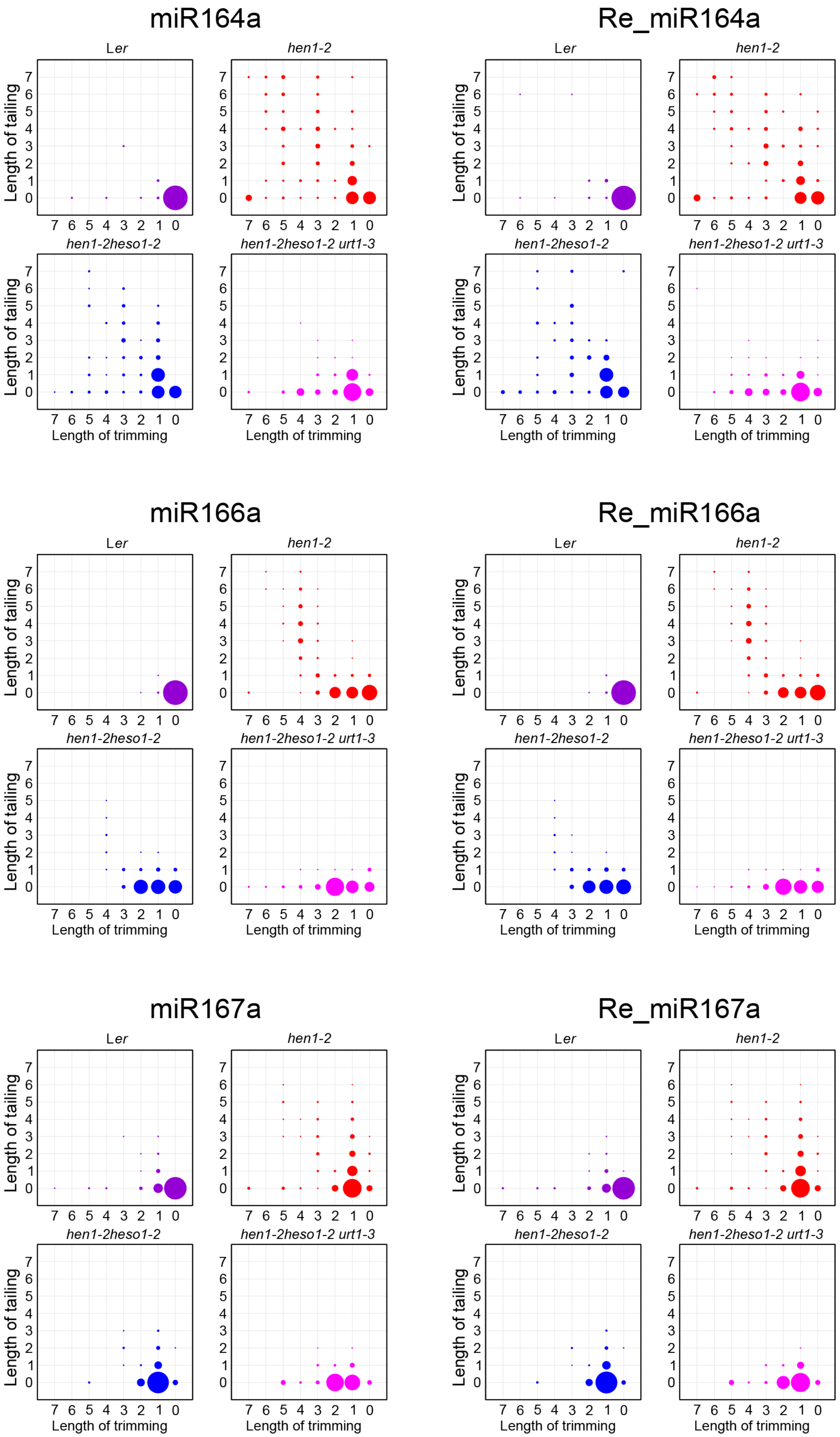
miR164a
Re_miR164a
miR166a
Re_miR166a
miR167a
Re_miR167a
Ler
hen1-2
hen1-2heso1-2
hen1-2heso1-2 urt1-3
Length of tailing
Length of trimming
7 6 5 4 3 2 1 0

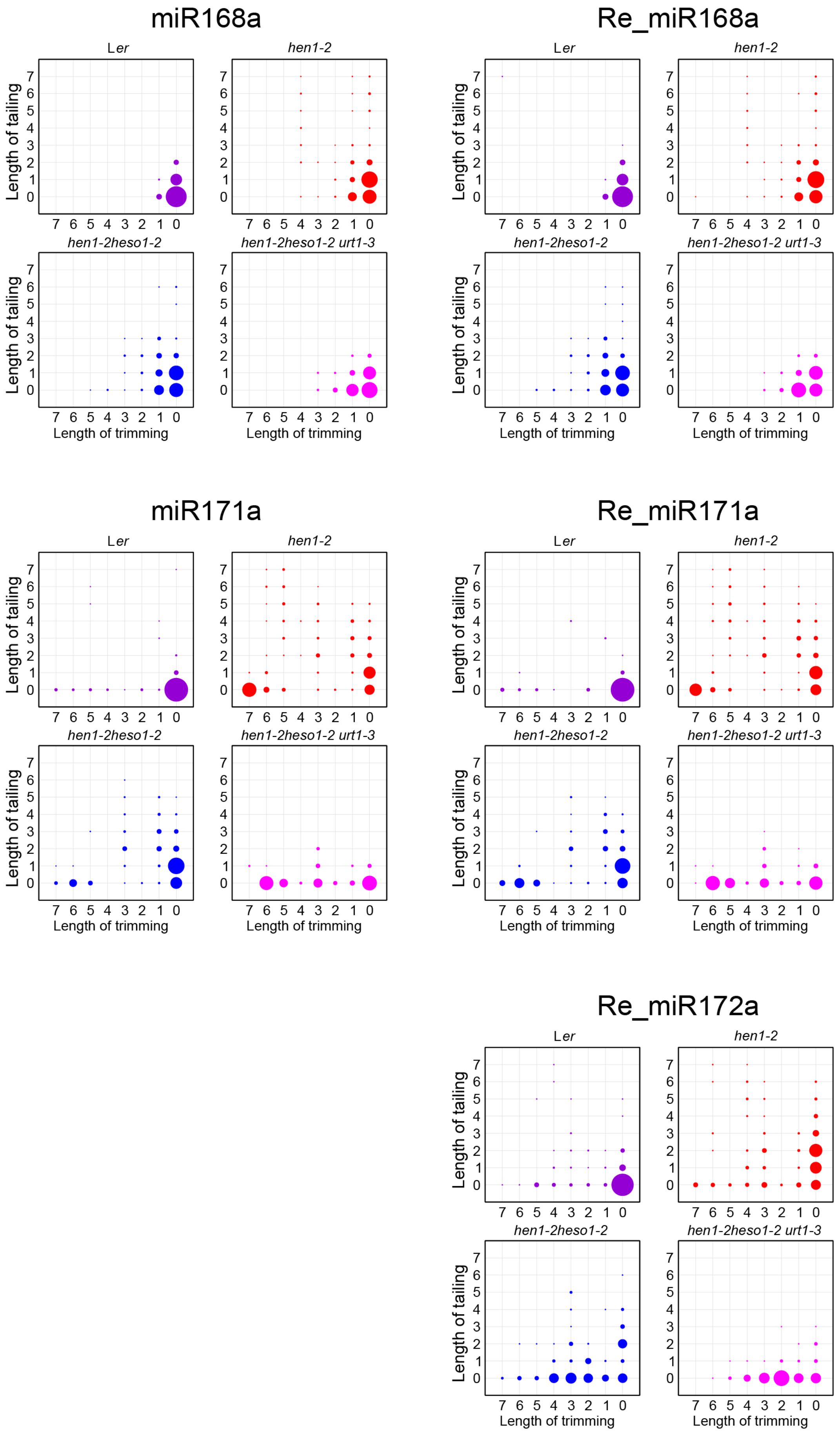
miR168a
Re_miR168a
miR171a
Re_miR171a
Re_miR172a
Ler
hen1-2
hen1-2heso1-2
hen1-2heso1-2 urt1-3
Length of tailing
Length of trimming

miR173

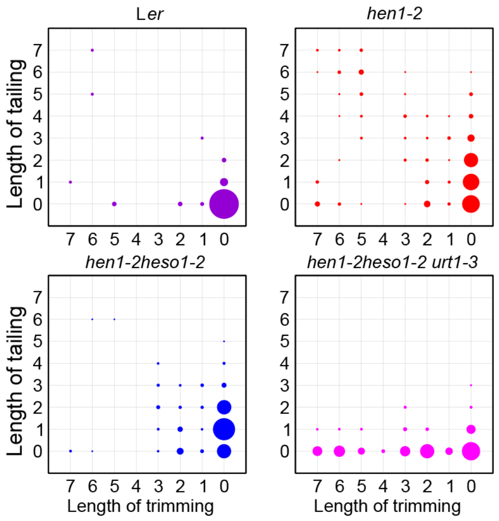

Re_miR173

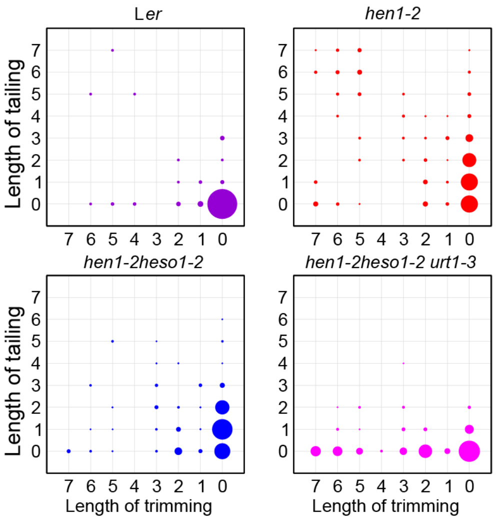

miR319a

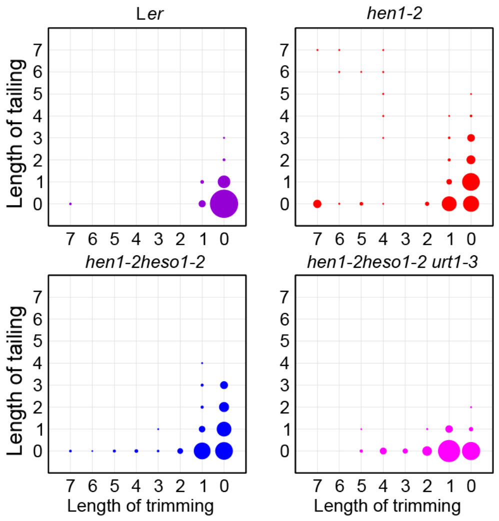

Re_miR319a

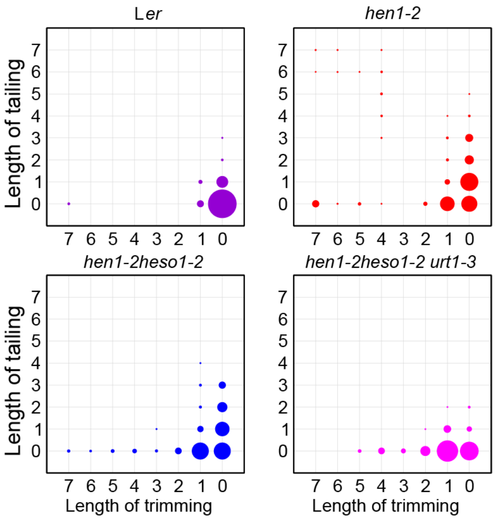

miR390a

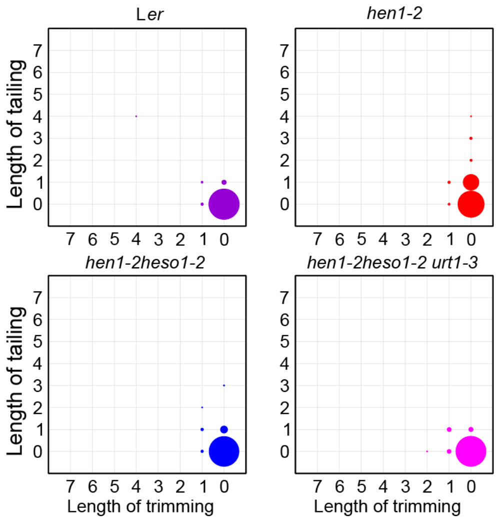

Re_miR390a

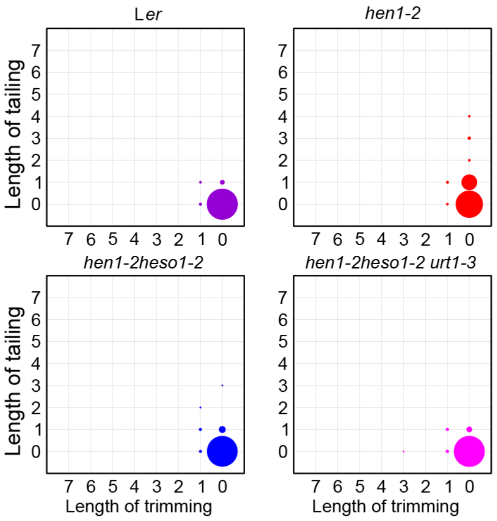

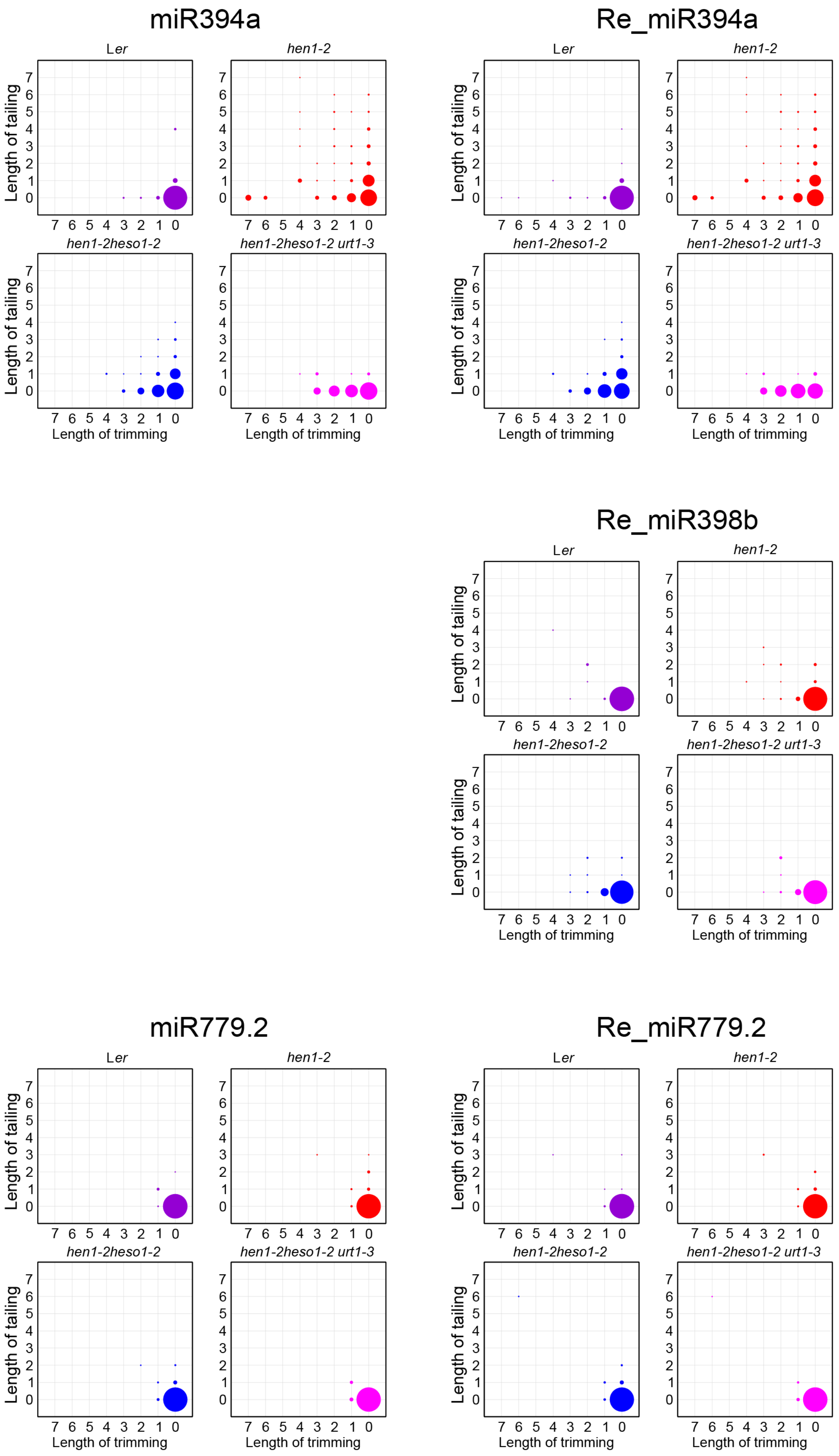
miR394a
Re_miR394a
Re_miR398b
miR779.2
Re_miR779.2
Ler
hen1-2
hen1-2heso1-2
hen1-2heso1-2 urt1-3
Length of tailing
Length of trimming
7 6 5 4 3 2 1 0

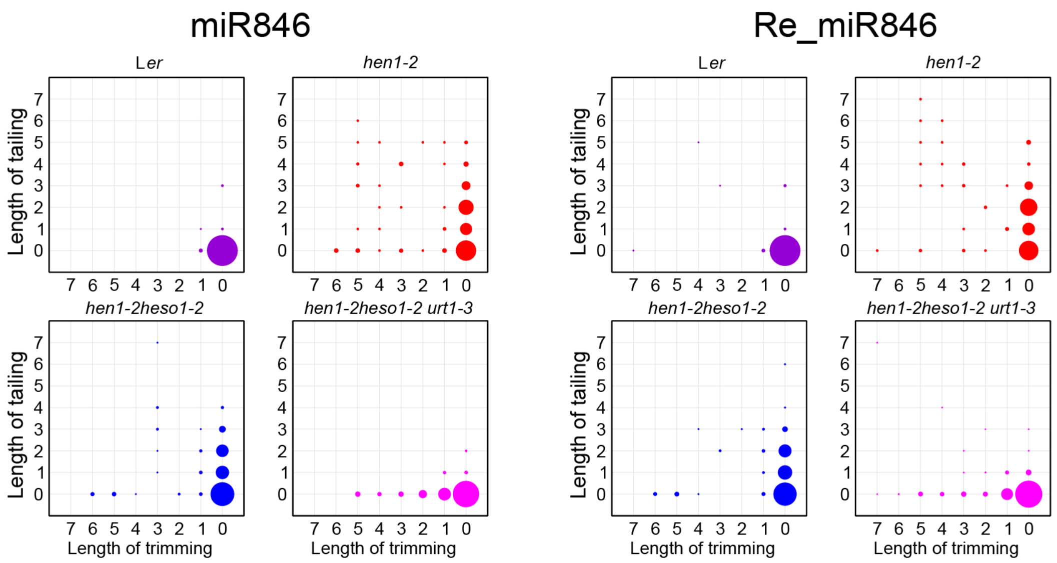

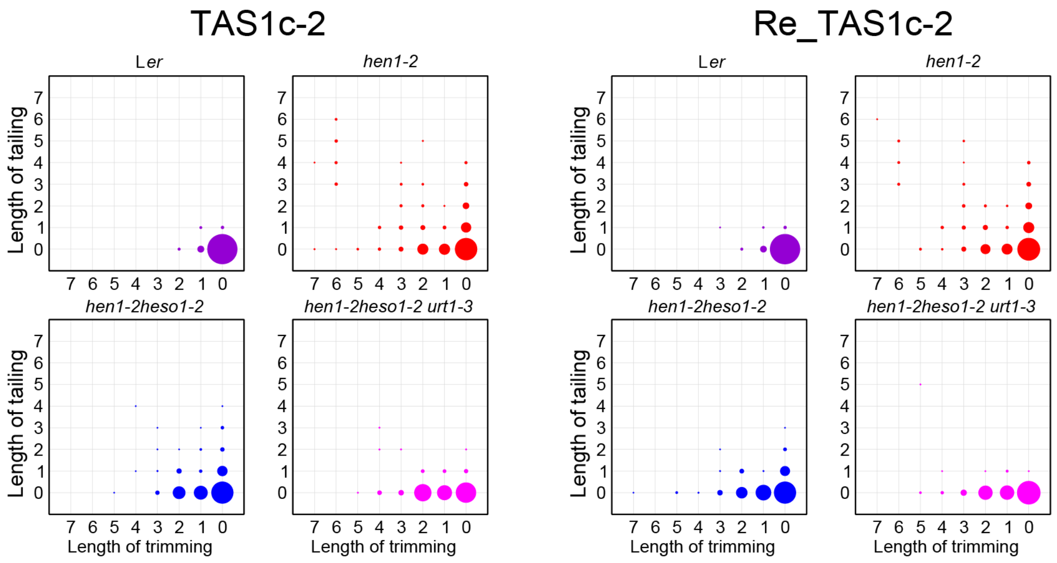

TAS1c-2: TCCAATGTCTTTTCTAGTTCGT

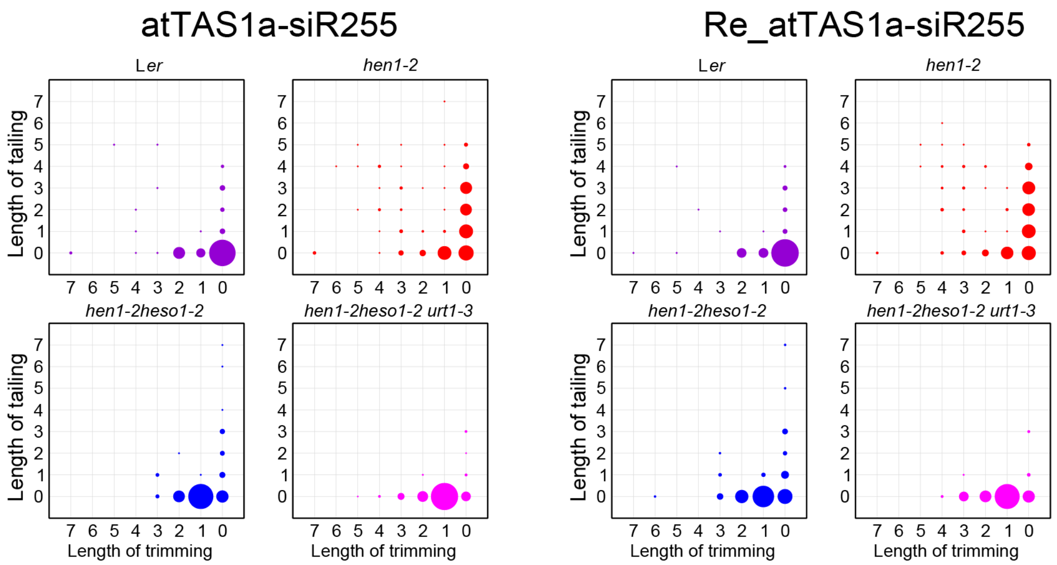

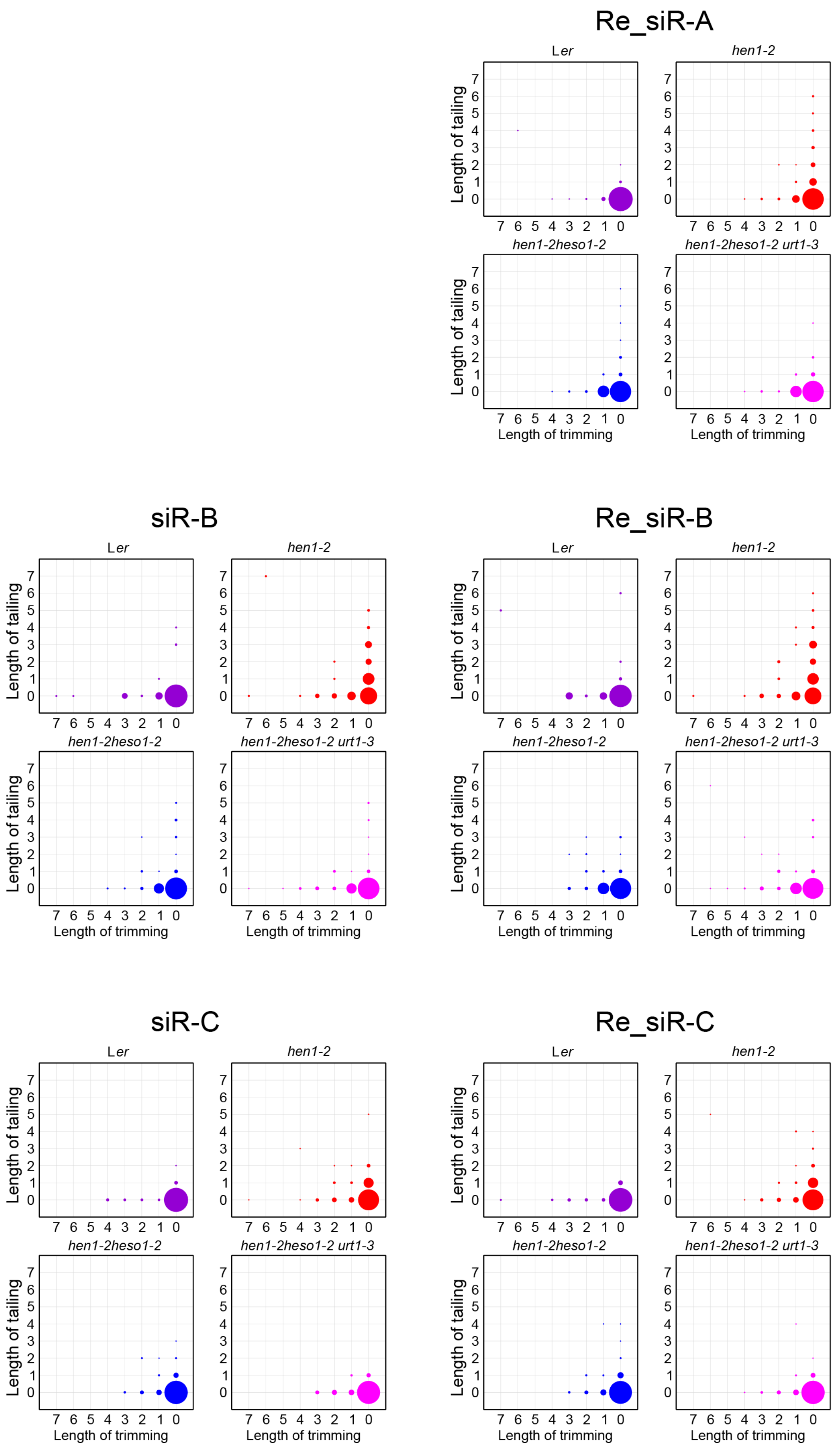
Re_siR-A
Ler
hen1-2
hen1-2heso1-2
hen1-2heso1-2 urt1-3
Length of tailing
Length of trimming
siR-B
Re_siR-B
siR-C
Re_siR-C
