# Supplementary material for: Synergistic and Independent Actions of Multiple Terminal Nucleotidyl Transferases in the 3’ Tailing of Small RNAs in Arabidopsis
Source: PLoS Genet. 2015 Apr 30;11(4):e1005091. doi: 10.1371/journal.pgen.1005091 (PMC4415790; doi:10.1371/journal.pgen.1005091)
Supplement: S3 Table — Note that only HESO1 and URT1 possess all three signatures. (PDF) [file pgen.1005091.s009.pdf]

S3 Table

	PAP	PAP associated	Linker region and P-motif
HESO1	YES	YES	YES
URT1	YES	YES	YES
At3g56320	YES	YES	NO
At5g53770	YES	YES	NO
At3g51620	YES	YES	NO
At2g40520	YES	YES	NO
At4g00060	YES	YES	NO
At3g45750	YES	NO	YES
At3g45760	YES	NO	YES
At3g61690	YES	NO	NO
