# Supplementary material for: Synergistic and Independent Actions of Multiple Terminal Nucleotidyl Transferases in the 3’ Tailing of Small RNAs in Arabidopsis
Source: PLoS Genet. 2015 Apr 30;11(4):e1005091. doi: 10.1371/journal.pgen.1005091 (PMC4415790; doi:10.1371/journal.pgen.1005091)
Supplement: S4 Table — (DOCX) [file pgen.1005091.s010.docx]

**S4 Table** List of primers and oligos in this study.

| Name | Sequence (5’ – 3’) | Applications |
| --- | --- | --- |
| **Small RNA probes** | | |
| miR166 | GG+GGA+ATG+AAG+CCT+GGT+CCG+T | Northern blot |
| miR167 | T+AGA+TCA+TGT+TGG+CAG+TTT+CA | Northern blot |
| miR171 | G+ATA+TTG+GCG+CGG+CTC+AAT+CA | Northern blot |
| miR172 | AT+GCA+GCA+TCA+TCA+AGA+TTC+T | Northern blot |
| U6 | TCATCCTTGCGCAGGGGCCA | Northern blot |
| **Primers for qRT-PCR** | | |
| CUC2qF | GCACCAACACAACCGTCACAG | qRT-PCR |
| CUC2qR | GAATGAGTTAACGTCTAAGCCCAAGG |  |
| CUC1qF | CGCCTTGACGGCAAATTCTCTTAC | qRT-PCR |
| CUC1qR | GATGATCGGAGCAATTGCAGAACC |  |
| HAP2bqF | TGCTGCAATTTCAAAACCTG | qRT-PCR |
| HAP2bqR | TCACCTCACGGCGAAGTTAC |  |
| AGO1qF | AAGGAGGTCGAGGAGGGTATGG | qRT-PCR |
| AGO1qR | CAAATTGCTGAGCCAGAACAGTAGG |  |
| ACT2-RT-F | CTCTCCGCTTTGAATTGTCTCGTTG | qRT-PCR |
| ACT2-RT-R | GGTACCATTGTCACACACGATTGGT |  |
| SPL10qF | GTGGGAGAATGCTCAGGAGGC | qRT-PCR |
| SPL10qR | GAGTGTGTTTGATCCCTTGTGAATCC |  |
| GAPDHqF | TCTTTCCC TGCTCAATGCTCCTC | qRT-PCR |
| GAPDHqR | TTTCGCCACTGTCTCTC CTCTAAC |  |
| **Plasmid Construction** | | |
| URT1gGWF | CACCTCAACATAACAATCAACCCCAACG | Complementation |
| URT1gGWR | GTTGTGGCCTTGTCCATTATTATCTTC |  |
| URT1cGWF | CACCATGGCGGACGGTGGGGCTGAAC | Overexpression |
| URT1cGWR | GTTGTGGCCTTGTCCATTATTATCTTC |  |
| GST-URT1F | CATCTCGAGATGGCGGACGGTGGGGCTGAAC | Prokaryotic expression |
| GST-URT1R | CATCTCGAGTCAGTTGTGGCCTTGTCCATTA |  |
| **Genotyping** | | |
| heso1-2dCAPSF | AGATGTTCTATGTTGTTTGCTGCA | *heso1-2* genotyping |
| heso1-2dCAPSR | ATATTTTGTGTGGTTTGGCTTCA |  |
| urt1-3dCAPSF | gaagtgaagttactttggattgca | *urt1-3* genotyping |
| urt1-3dCAPSR | gttattatacacacCTGTAAGCTA |  |
| **RNA oligo** | | |
| miR166a | TCGGACCAGGCTTCATTCCCC | RNA ligation |
